# Sucrose triggers a novel signaling cascade promoting *Bacillus subtilis* rhizosphere colonization

**DOI:** 10.1038/s41396-021-00966-2

**Published:** 2021-03-26

**Authors:** Tao Tian, Bingbing Sun, Haowen Shi, Tantan Gao, Yinghao He, Yan Li, Yixue Liu, Xuexian Li, Liqun Zhang, Shidong Li, Qi Wang, Yunrong Chai

**Affiliations:** 1 Institute of Plant Protection, Tianjin Academy of Agricultural Sciences, Tianjin, China; 2 Department of Plant Pathology, China Agricultural University, Beijing, China; 3 Department of Biology, Northeastern University, Boston, MA USA; 4 Department of Plant Nutrition, China Agricultural University, Beijing, China; 5 Institute of Plant Protection, Chinese Academy of Agricultural Sciences, Beijing, China

**Keywords:** Soil microbiology, Bacteriology

## Abstract

Beneficial rhizobacteria promote plant growth and protect plants against phytopathogens. Effective colonization on plant roots is critical for the rhizobacteria to exert beneficial activities. How bacteria migrate swiftly in the soil of semisolid or solid nature remains unclear. Here we report that sucrose, a disaccharide ubiquitously deployed by photosynthetic plants for fixed carbon transport and storage, and abundantly secreted from plant roots, promotes solid surface motility (SSM) and root colonization by *Bacillus subtilis* through a previously uncharacterized mechanism. Sucrose induces robust SSM by triggering a signaling cascade, first through extracellular synthesis of polymeric levan, which in turn stimulates strong production of surfactin and hyper-flagellation of the cells. *B. subtilis* poorly colonizes the roots of *Arabidopsis thaliana* mutants deficient in root-exudation of sucrose, while exogenously added sucrose selectively shapes the rhizomicrobiome associated with the tomato plant roots, promoting specifically bacilli and pseudomonad. We propose that sucrose activates a signaling cascade to trigger SSM and promote rhizosphere colonization by *B. subtilis*. Our findings also suggest a practicable approach to boost prevalence of beneficial *Bacillus* species in plant protection.

## Introduction

The soil-dwelling *Bacillus subtilis* is an excellent biological control agent, capable of suppressing a number of soil-borne phytopathogens [1, 2]. Evidence suggests that colonization on plant roots and formation of root-associated biofilms are critical for *B. subtilis* to exert biocontrol activities [3–5]. Rhizosphere, influenced by root-released nutrients, is a relatively eutrophic ecological micro-niches [6–9]. It is well established that plants actively recruit beneficial microorganisms and shape the rhizomicrobiome [6, 10–13]. Considering the relatively large proportion and the diverse and dynamic nature of root exudates among the nutrients released by the plant, root exudates likely play an important role in promoting bacterial root colonization and shaping the rhizomicrobiome [14, 15].

As one of the most important biological control agents (BCAs), how *B. subtilis* overcomes the obstacle of distance to reach the rhizoplane, and establishes beneficial interactions with the roots remains not well understood [16–18]. Chemotaxis, a directed bacterial swimming propelled by flagella in aqueous environments, was shown to play an indispensable role in the colonization of *B. subtilis* on the *Arabidopsis thaliana* roots [19, 20]. Meanwhile, swarming motility was proposed as a more practicable mechanism for the translocation of cells in the natural semiarid soil [21–24]. Swarming on the semisolid surface is characterized by hyper-flagellation of cells and strictly depends on surfactin [22, 25, 26]. Recent studies suggest that swarming plays a critical role in the migration of *B. subtilis* cells to the rhizoplane [27, 28]. Yet environmental signals triggering swarming in *B. subtilis* are largely unknown [21, 29]. It is worth pointing out that most studies would suggest that *B. subtilis* cells do not swarm on solid surface. An unsettled question thus arising is how *B. subtilis* cells manage to efficiently migrate in the semisolid or solid soil environment [30, 31].

We started by investigating root-secreted chemicals that could potentially promote *B. subtilis* root colonization. Here we report that sucrose, a disaccharide deployed by virtually all photosynthetic plants for fixed carbon transport and storage and abundantly released from the roots [32, 33], promotes solid surface motility (SSM) and root colonization by *B. subtilis* through a previously uncharacterized mechanism. Sucrose induces robust SSM of *B. subtilis* by triggering a signaling cascade via a so-called “levan detour”. We further discuss the role of sucrose in establishing symbiosis between *B. subtilis* and plant roots.

## Results

### Sucrose promotes root colonization and induces solid surface motility (SSM) by *B. subtilis*

**Fig. 1:**
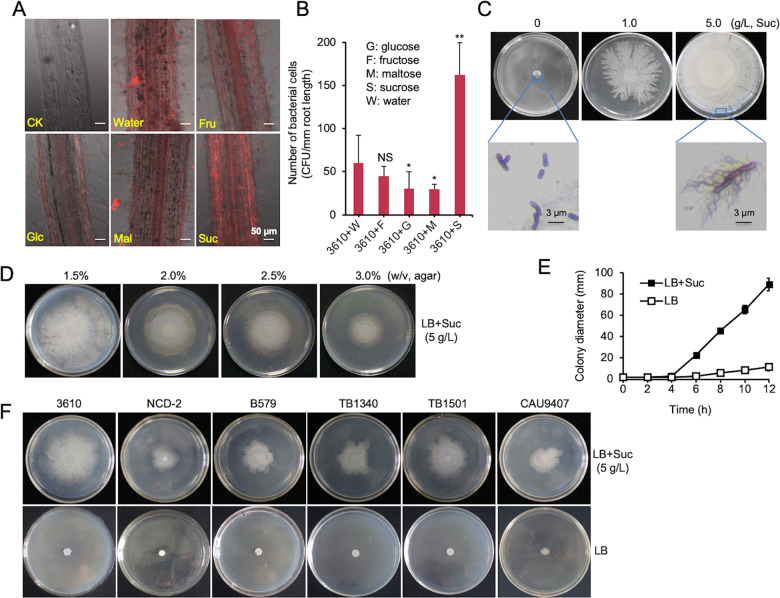
Sucrose promotes root colonization and solid surface motility (SSM) by *B. subtilis*. **A** LSCM images of 18-day-old tomato roots observed 72 h after inoculation with a *B. subtilis* 3610 derivative constitutively expressing *mKate2* and treated with different sugars (Fru fructose, Glc glucose, Mal maltose, and Suc sucrose). For treatments of sugars, 5 ml cell suspension supplemented with 0.5% (w/v) sugar was applied to the roots. Water indicates the same volume of water replacing any sugar solution. CK indicates no addition of bacterial cells and sugars. Shown pictures are representatives of at least 20 independent root samples (scale bars: 50 μm). **B** The influence of different sugar supplementations on the colonization of 3610 cells on tomato roots was determined by counting colony forming unit (CFU) per mm root length. Error bars represent standard deviations. * indicates *p* value < 0.05; ** indicates *p* value < 0.01; NS no statistical difference. **C** The concentration of supplementary sucrose positively correlated with the robustness of solid-surface movement (SSM) by 3610. The microscopic images of outer-edge cells stained with flagella-specific dye, on solid LB plate with or without sucrose (5 g/L). Pictures are representatives of at least five independent samples (scale bars: 3 μm). **D** The increasing concentrations of agar negatively influenced the robustness of sucrose-induced SSM. **E** Colony expanding rate of 3610 on solid LB plate with or without supplementary sucrose (5 g/L) was determined by measuring the diameter of the colony periodically. Assays were done in triplicate. Error bars represent standard deviations. **F** Addition of sucrose (5 g/L) also triggered SSM by other *Bacillus* strains on solid LB plates. All Petra dishes shown here have a diameter of 10 cm.

We previously revealed that both root-released malic acid and complex polysaccharides from plants induce formation of root-associated biofilms by *B. subtilis* [4, 5]. Here we investigated the influence of root-released simple sugars (mono- and di-saccharides) on root colonization by *B. subtilis*. We employed a red fluorescence-labeled *B. subtilis* strain (YC843, a derivative of the model strain NCIB 3610) to study the influence of selected sugars on bacterial colonization on tomato roots in the sterilized soil. Images from laser scanning confocal microscope (LSCM) suggested more robust root colonization by *B. subtilis* in the presence of sucrose than several other root-secreted sugars (Fig. 1A). Plate recovery counting confirmed that sucrose boosted *B. subtilis* root colonization by about 2.7-fold compared to no sugar addition, an effect not seen in either glucose, fructose, or maltose (Fig. 1B). To test the possibility that the enhanced root colonization is due to the effect of sucrose on *B. subtilis* growth, we performed assays of growth of *B. subtilis* cells in two minimal media (MSgg [34] and M9 [35]) as well as LB broth supplemented with either sucrose or any other sugars (5 g/L). None of the supplemented sugars, had a significant impact on the growth of *B. subtilis* cells while addition of fructose caused a slight reduction in the final cell density of the minimal media cultures (Fig. S1A–C). Thus, our results suggest a previously uncharacterized role of sucrose in promoting root colonization by *B. subtilis*.

When spotted on solid LB agar plates (1.5% agar, w/v), we noticed that sucrose triggered a robust swarming-like motility in the *B. subtilis* strain NCIB 3610 (Fig. 1C, hereafter referred to as 3610). The velocity of this motility correlated positively with the quantity of supplemented sucrose (Fig. 1C), but negatively with the concentration of agar (Fig. 1D). The maximal speed of the bacterial movement on the solid LB plates supplemented with sucrose (5 g/L) is estimated to be ~0.6 cm/h at 37 °C (Fig. 1E). Cells at the outer-edge of the colony presented a hyper-flagellated phenotype (Fig. 1C). This sucrose-induced SSM depends on the flagellar since a flagellar deficient mutant (Δ*hag*) completely failed to perform SSM (Fig. S2A). This SSM was also observed in other *Bacillus* strains, including *B. subtilis* (B579, NCD-2, and CAU9407) and *B. velezensis* (TB1340 and TB1501) (Figs. 1F, S3, Table S1). Interestingly, the observed SSM was less robust in other *Bacillus* strains than in 3610, indicating that subtle strain differences may have an impact on the intensity of the SSM. Since swarming motility by *Bacillus* is often studied in semisolid media (0.6–0.7% agar) [26], this sucrose-induced SSM could represent a novel motility behavior. Alternatively, it suggests that swarming could occur even on solid surface under certain natural circumstances in some *Bacillus* species. Considering the semisolid or solid nature of soil particles and even the root surface, we suspect that this novel SSM could play a role in the migration of *B. subtilis* cells from bulk soil to rhizosphere and even along the rhizoplane.

### Sucrose induces strong surfactin production

**Fig. 2:**
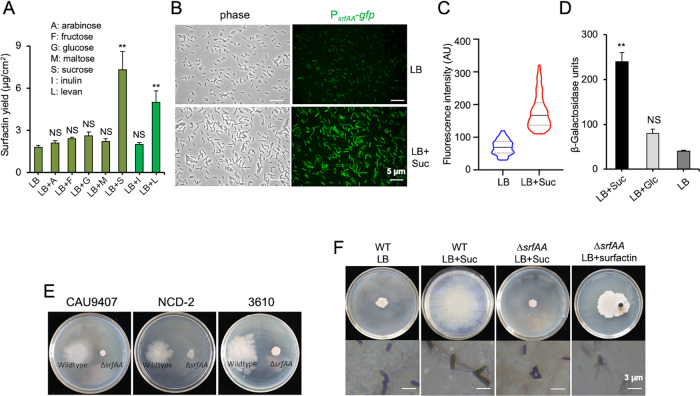
Surfactin mediates sucrose-induced SSM by *B*. subtilis. **A** The effect of various sugars on surfactin yield (µg/cm^2^) by *B. subtilis* 3610 cells was assayed on solid LB agar plates (1.5% agar, w/v). Samples were collected from the plates supplemented with different sugars, all at the concentration of 5 g/L. Surfactin was extracted, and the amount of surfactin was determined by HPLC as described in the method. The error bars represent standard deviations from triplicate assays. * indicates *p* value < 0.05; ** indicates *p* value < 0.01; NS no statistical difference. **B** Microscopy images of cells harboring the promoter fusion (P_*srfAA*_-*gfp*, CY106) collected from the edge of the colonies after 4 h of inoculation on solid LB plates with or without 5 g/L sucrose (scale bars: 5 μm). **C** Quantification of fluorescence intensity of the cells expressing P_*srfAA*_-*gfp* from above. The quantification for each sample is based on roughly 200 cells by using ImageJ. Solid lines in the middle indicates the mean value (artificial units, AU) of the fluorescence intensity. Upper and lower dotted lines indicate the 75% and 25% quartile, respectively. **D** Assays of β-galactosidase activities of cells bearing the P_*srfAA*_-*lacZ* promoter fusion (KG203). Cells were similarly collected from the edge of the colonies after 4 h inoculation from solid LB agar plates without sugar addition, with the addition of 5 g/L sucrose or glucose. Assays were done in biological triplicates. Error bars represent standard deviations. ** indicates *p* value < 0.01; NS, no statistical difference. **E** The *srfAA* mutants of three *B. subtilis* strains (9407, NCD-2, and 3610) lost SSM on solid LB with 5 g/L sucrose plates when compared to their wild-type counterparts. Images are representatives of at least 3 independent assays. **F** Pure surfactin, but not sucrose, rescues the phenotype of SSM and hyper-flagellation in a *srfAA* mutant of 3610 (Tm01). Solutions (in a volume of 100 μL) containing 180 μg surfactin were filled into an Oxford-cup, which is 1 cm away from the inoculating spot with the *B. subtilis* cells. All Petra dishes shown here have a diameter of 10 cm.

Swarming by *B. subtilis* strictly depends on surfactin production [22, 25, 26]. It was conceivable that sucrose might stimulate strong production of surfactin when triggering SSM. Hence, the influence of sucrose on surfactin production by *B. subtilis* was determined. We performed assays of surfactin production by *B. subtilis* in both LB and the minimal medium MSgg. On solid LB agar plates supplemented with sucrose, cells had an about fourfold increase in surfactin production when compared to cells on just solid LB without sucrose, while other tested sugars (arabinose, fructose, glucose, and maltose) only registered a slight increase in surfactin yield (Fig. 2A). On MSgg agar plates, the overall surfactin yield was modestly lower, yet the addition of sucrose increased surfactin production by more than threefold (Fig. S4B). We next tested if sucrose enhances surfactin production by inducing the *srf* operon, which encodes the enzyme complex for surfactin synthesis [36, 37]. We first employed a reporter strain of 3610 harboring a *gfp* gene fused to the *srf* promoter (P_*srfAA*_-*gfp*, CY106). We collected cells after 4 h incubation on solid LB plates with or without supplementation of sucrose and examined them under fluorescent microscopy. A much brighter fluorescence was observed for cells harvested from the plates supplemented with sucrose than no sucrose (Fig. 2B). Quantification of the fluorescence signal density indicates about 2.7-fold increase in cells treated with sucrose than without sucrose (Fig. 2C). In another experiment, we compared the activity of the *srf* operon in the presence or absence of sucrose by using a *B. subtilis* strain bearing the P_*srfAA*_-*lacZ* reporter (KG203). We performed assays of β-galactosidase activities on cells similarly collected from solid LB plates without or with sucrose supplementation, or with supplementation of glucose (5 g/L). Addition of sucrose stimulated the activity of the P_*srfAA*_-*lacZ* reporter by about 5.9-fold (Fig. 2D), consistent with the above results from the P_*srfAA*_-*gfp* reporter (Fig. 2C). Addition of glucose also stimulated the reporter activity, albeit at a much milder level (Fig. 2D), which could be due to altered metabolic regulation by glucose. Our results indicate that sucrose enhances surfactin production through gene induction.

To confirm phenotypically that sucrose-induced SSM depends on strong surfactin production, we tested the influence of sucrose on a ∆*srfAA* mutant of 3610 (Tm01) deficient in surfactin production [38]. The ∆*srfAA* mutant completely lost motility on solid LB plates even with sucrose supplementation (far right-hand, Fig. 2E), as well as the ∆*srfAA* mutants of two closely related *B. subtilis* strains (9407 and NCD-2). Interestingly, the loss of SSM and hyper-flagellation phenotype of the ∆*srfAA* mutant could be rescued by exogenously supplementing surfactin even in the absence of sucrose (spotted 1 cm away from the ∆*srfAA* colony on LB, Fig. 2F). These results provide further evidence that sucrose-induced SSM depends on strong surfactin production. Whether surfactin serves solely as a biosurfactant to facilitate surface migration of cells or serves as a signal as well is not known. However, the induction of hyper-flagellation of the ∆*srfAA* cells by surfactin suggests its potential role as a signal (Fig. 2F). Our result here is also consistent with previous reports that surfactin induces synthesis of flagellar genes in *B. subtilis* [39] and that sucrose is able to induce surfactin production in a closely related *B. amyloliquefaciens* strain [40].

### Sucrose induces SSM via “a levan detour”

**Fig. 3:**
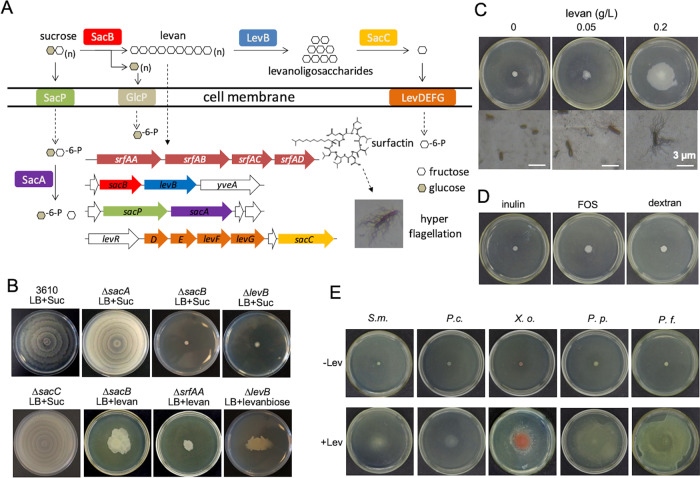
Levan induces SSM in *B. subtilis* and other soil bacteria. **A** A schematic diagram of sucrose metabolism and the signal relay triggering SSM in *B. subtilis*. On the left of the diagram, it indicates that the extracellular sucrose is imported into cells by the SacP transporter, and then hydrolyzed into glucose-6-P and fructose by the SacA hydrolase. On the top, it illustrates that sucrose is metabolized extracellularly. The levansucrase SacB uses sucrose to synthesize polymeric fructoses (levan), and glucose. When needed, levan can be degraded into levanoligosaccharides by LevB, and into monomeric fructoses by SacC, which is then imported into cells by the transporter composed of LevDEFG. Levan likely indirectly actives the *srfAA-AD* operon, whose product (surfactin) triggers SSM in *B. subtilis*. **B** SSM by *B. subtilis* 3610 and various mutants on solid LB plates supplemented with sucrose (5 g/L), levan (0.2 g/L), or levanbiose (0.2 g/L). Pictures are representatives of at least three independent assays. **C** Pure levan, when supplied at 0.2 g/L, induces SSM and hyper-flagellation in *B. subtilis* 3610. **D** The structural analogues of levan (inulin, FOS, and dextran) cannot induce SSM by *B. subtilis* 3610 when provided at 0.2 g/L. **E** Levan (0.2 g/L) induces SSM by some soil bacteria (*Serratia marcescens* T4-3, *Pectobacterium carotovorum* subsp. carotovorum Z3-3, *Xanthomonas oryzae* pv. oryzae PXO99F, *Pseudomonas protegens* pf-5, and *P. fluorescens* 2p24). All pictures are representatives of at least three independent assays. All Petra dishes shown here have a diameter of 10 cm.

Sucrose can be taken into the cells via the phosphor-transferase system (PTS) encoded by the *sacP* gene and hydrolyzed to glucose-6-P and fructose by a sucrose hydrolase encoded by the highly conserved *sacA* gene in *B. subtilis* [41, 42] (Fig. 3A). However, neither glucose nor fructose induced SSM in 3610 (Fig. S2B). Further, a ∆*sacA* mutant of 3610 (Tm13) retained the ability of SSM upon sucrose induction (Fig. 3B), indicating that the *sacA* pathway is not involved in the sucrose-induced SSM. Some *Bacillus* species employ another sucrose utilization pathway, in which a *sacB* gene encodes a levansucrase converting sucrose extracellularly to polymeric fructan (levan) and glucose [43] (Fig. 3A). To determine if the *sacB* pathway is involved in sucrose-induced SSM, we tested the SSM by the ∆*sacB* mutant. Surprisingly, the ∆*sacB* mutant (Tm14) completely lost the ability to perform SSM, strikingly different from the WT and ∆*sacA* (Fig. 3B). Furthermore, exogenously added levan (0.2 g/L) partially rescued the SSM deficiency of ∆*sacB* (Fig. 3B). Levan also induced SSM and hyper-flagellation by 3610 on solid LB plates without sucrose (Fig. 3C), and elevated surfactin production by about 2.3-fold (Fig. 2A). Three structural analogues of levan [inulin, fructo-oligosaccharide (FOS), and dextran] failed to induce SSM in the parallel experiment (Fig. 3D). Inulin also did not induce surfactin production (Fig. 2A). We reasoned that the difference in the fructose linkage between levan (β-2,6-linkage) and FOS and inulin (both β-2,1-linkage) might contribute to the divergent inducing effect. Lastly, we extended the test with several plant polysaccharides, including xylose, pectin, and cellulose; none of them showed noticeable inducing effects under the same conditions (Fig. S2C). These results collectively suggested a specific effect of levan in inducing SSM by *B. subtilis*.

We showed earlier that sucrose induces surfactin production (Figs. 2A, S4). To test if the induction is dependent on either one of the two sucrose utilization pathways (SacA or SacB, Fig. 3A), we compared surfactin production by cells of either the WT, or the Δ*sacA*, or Δ*sacB* mutant. The assays were performed in both LB and the minimal medium MSgg. Our results suggest that in both media, sucrose largely failed to induce surfactin production in the Δ*sacB* cells, but not the Δ*sacA* cells (Fig. S4A, B). These results reinforced the idea that levan induces surfactin production.

Levan can be further hydrolyzed into levanoligosaccharides by LevB, and into fructoses by SacC (Fig. 3A) [44]. Upon further test, we found that the *∆levB* mutant (Tm26), but not *∆sacC* (Tm23), also lost the ability to perform SSM (Fig. 3B), indicating the involvement of levanoligosaccharides in promoting SSM. In support of the above idea, the defective motility of *∆levB* can be partially rescued by addition of levanbioses, one of the degradative products of levan by LevB (Fig. 3B). The reason why the rescue effect by levanbioses is less robust than that of levan, we suspect, is because the breakdown products of levan by LevB are likely a mixture of various oligosaccharides including levanbioses [45], and we do not know yet which specific oligosaccharide is most potent in inducing SSM. We rephrased the SacB-LevB pathway as the “levan detour”. Previously, the potential metabolic benefit of extracellular production of levan by this *sacB* pathway was not as clear as the *sacA* pathway. The *sacB* pathway is also not as widely present in bacteria as the *sacA* pathway.

### The “levan detour” confers competitive advantages to *B. subtilis* during root colonization

**Fig. 4:**
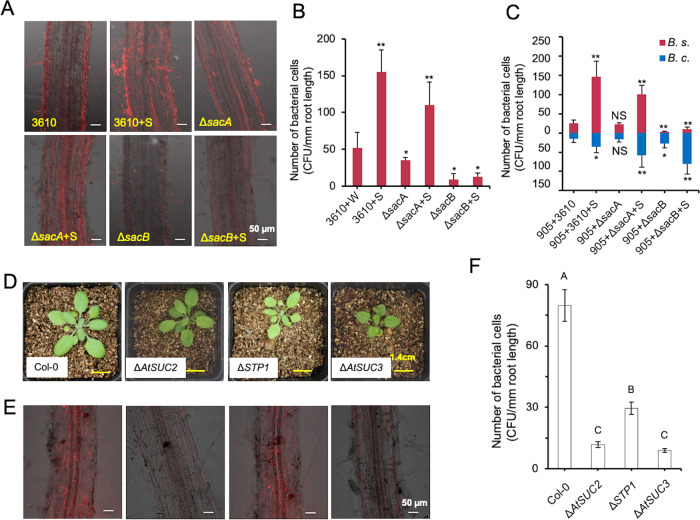
Sucrose promotes competitive root colonization by *B. subtilis*. **A** LSCM images of root colonization by WT (3610) and the mutants (Δ*sacA* and Δ*sacB*) treated without or with sucrose (+S). **B** The difference on the tomato root colonization between the WT and the two mutants was determined by counting colony forming unit (CFU) per mm root length. Error bars represent standard deviations. ** indicates *p* value < 0.01; * indicates *p* value < 0.05. **C** The colonization competition between 905 and 3610 (including its derivates) on tomato roots was determined by counting CFU per mm root length. * indicates *p* value < 0.05; ** indicates *p* value < 0.01; NS, no statistical difference. **D** The pictures of seedlings of wild-type *A. thaliana* (Col-0) and its derivatives impaired in sucrose transport [Δ*AtSUC2* (At1g22710, SALK_0038124), Δ*AtSTP1* (At1g11260, SALK_048848c), and Δ*AtSUC3* (At2g02860, SALK_077723)]. Weakened developmental effects were observed in some of the mutants. Pictures are representative of at least 20 independent plants (Scale bars: 1.4 cm). **E** LSCM pictures of 15-day-old roots of wild-type *A. thaliana* and the mutants. The roots were observed 72 h after inoculation with strain 3610 constitutively expressing mKate2. Pictures are representative of at least 20 independent roots (Scale bars: 50 μm). **F** The influence of different sugar transporters on the colonization of 3610 on the *A. thaliana* roots was determined using colony forming unit (CFU) per mm root length by plate recovery counting. The letters above the columns indicate statistically significant differences of different groups based on Student’s *t* test (*p* < 0.01).

Adding sucrose to the soil promoted the colonization of *B. subtilis* on tomato roots (Fig. 1A, B). We wondered if this is due to sucrose inducing SSM by *B. subtilis* in the rhizosphere via the “levan detour”. We thus compared root colonization between the red fluorescence-labeled wild-type strain (YC843), and the “levan detour” mutant ∆*sacB* (Tm32), and ∆*sacA* (Tm31), under the pot soil conditions. It was found that the ∆*sacB* mutant, but not the ∆*sacA* mutant, failed to colonize roots effectively as compared to the wild type, even with the addition of sucrose (Fig. 4A, B). Again, addition of sucrose did not impact the growth of either ∆*sacA* or ∆*sacB* cells in either the minimal media (MSgg and M9) or LB media (Fig. S1E, F). These results indicate that not just sucrose, but the “levan detour” is indeed important for root colonization by *B. subtilis*.

To further support the idea that it is sucrose-induced motility but not sucrose metabolism promoting enhanced root colonization, we repeated tomato plant root colonization experiment in the presence or absence of sucrose using two motility-deficient mutants, ∆*hag* and ∆*srfAA*. The ∆*hag* mutant is expected to completely deficient in both swimming and swarming motility due to lack of flagellar while the ∆*srfAA* mutant is known to be deficient in swarming motility, but not swimming and chemotaxis in aqueous environments, due to loss of surfactin production. Our results showed that the ∆*hag* mutant was severely impaired in tomato root colonization performed under pot soil conditions even in the presence of sucrose (Fig. S5). The root colonization by the ∆*srfAA* mutant was also clearly impaired both in the presence and absence of sucrose, but surprisingly at a much lesser degree compared to the ∆*hag* mutant. This was not entirely unexpected since the ∆*srfAA* mutant still retained the ability of swimming and chemotaxis in aqueous environments (such as during initial bacterial inoculation and periodical watering during plant growth).

Lastly, we also tested if sucrose and levan can confer competitive advantages to *B. subtilis* during root colonization by co-inoculating both *B. subtilis* 3610 and *B. cereus* 905, another soil bacterium that lacks *sacB* [46]. In the absence of sucrose, there was only a modest difference in root colonization between 3610 and 905 as judged by CFU counting (3160 + 905, Fig. 4C). Addition of sucrose boosted root colonization by both 3610 and 905, but the increase was far more substantial in 3610 than in 905 (3610 + 905 + S). This substantial increase was reversed when the ∆*sacB* mutant of 3610 was co-inoculated with 905, with or without addition of sucrose; the recovery ratio of 905:*∆sacB* was at about 5:1 without sucrose, and up to 10:1 with sucrose supplemented (Fig. 4C). Meanwhile, the ∆*sacA* mutant of 3610 behaved very much like the wild type in the co-colonization experiment (905 + ∆*sacA*, Fig. 4C). Based on these results, we conclude that the “levan detour” confers significant competitive advantages to *B. subtilis* during root co-colonization.

### Plant sucrose secretion is critical for *B. subtilis* root colonization

More than 20% of photoassimilates are secreted into the soil by plant roots including a significant proportion of sucrose [8, 47]. Given the strong influence of exogenously added sucrose on root colonization by 3610, we reasoned that the endogenous, root-released sucrose could stimulate the colonization of 3610 *in planta*. In plants, sucrose/proton symporters mediate sucrose allocation towards sink tissues [48, 49]. In *Arabidopsis thaliana*, SUC2 serves as a predominant transporter in sucrose transport in phloem [48], while SUC3 is the major transporter involved in sucrose exudation from roots [50]. We decided to test root colonization of 3610 in WT and several sugar transport mutants of *A. thaliana* (Fig. 4D). As shown, *B. subtilis* had substantially lower root colonization efficiency toward *A. thaliana* Δ*suc2* and Δ*suc3* mutants as compared to the wild type (Col-0) and the general monosaccharide transporter mutant *STP1* [51, 52] (Fig. 4E, F). Plate recovery counting revealed approximately 6.8- and 8.9-fold decrease in the colonization efficiencies on the Δ*suc2* and Δ*suc3* mutants, respectively, compared to the wild type (Fig. 4F). These results support the idea that root exudation of sucrose plays a pivotal role in root colonization by *B. subtilis*.

### Sucrose selectively shapes the rhizomicrobiome and enhances disease control

**Fig. 5:**
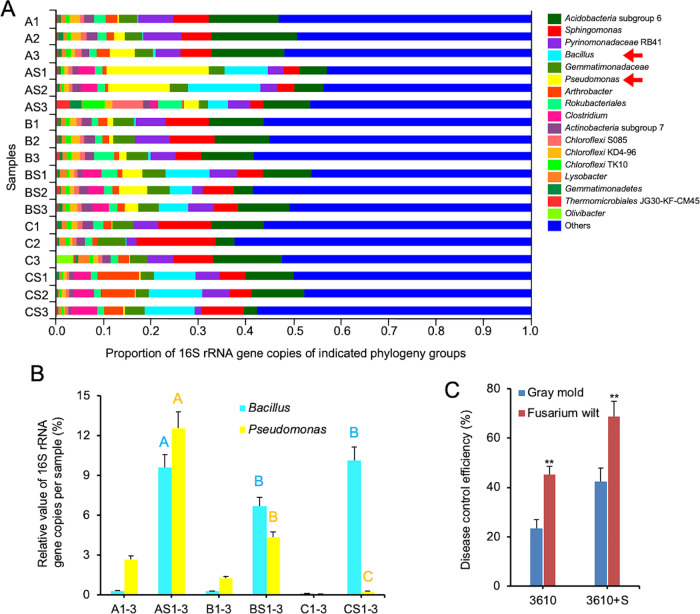
Sucrose selectively shapes rhizomicrobiome and enhances disease control by *B. subtilis*. **A** The relative abundance of 18 top genus groups. Community barplot analysis of bacterial taxonomic groups (genus level) in the tomato root rhizosphere with *B. subtilis* 3610 or Δ*sacB*, and with or without the addition of sucrose. Different treatments are marked as: A (WT), AS (WT + sucrose), B (Δ*sacB*), BS (Δ*sacB* + sucrose), C (-sucrose), and CS (+sucrose). The numbers 1–3 represent three repetitions. The 18 different colors represent different bacterial genus. The *Bacillus* and *Pseudomonas* groups are highlighted by arrows. **B** The ratio of *Bacillus* and *Pseudomonas* species in the microbiome samples based on the relative value of averaged 16S rRNA gene copies per sample (16S rRNA copies/g soil) in the rhizomicrobiome samples with *B. subtilis* 3610 or Δ*sacB* mutant in sucrose and no sucrose conditions. Note: A *B. subtilis* 3610, AS *B. subtilis* 3610 plus sucrose, B Δ*sacB* mutant, BS Δ*sacB* mutant plus sucrose, C no 3610 and sucrose, and CS with only sucrose. Values are given as means of three independent biological replicates and the bars represent the standard error. The letters above the columns indicate statistically significant differences based on the 16S rRNA gene copies of *Bacillus* or *Pseudomonas* per sample and using the Student’s *t* test (*p* < 0.01). **C** Combination of sucrose and the *B. subtilis* improved the biological control efficiency against the soil-borne disease (fusarium wilt) and the resistance to the air-borne disease (gray mold) in tomato. ** indicates *p* value < 0.01; NS no statistical difference. The error bars represent standard deviations from triplicate assays.

To investigate if and how root-released sucrose broadly impacts rhizomicrobiome, 16S rRNA gene MiSeq sequencing was performed using samples associated with tomato roots pot-grown in natural soil. The microbiome samples were subjected to treatments with or without sucrose supplementation as well as with or without inoculation of *B. subtilis* (WT or Δ*sacB*), constituting a total of 6 different treatments (Fig. S6). 16S rRNA gene sequencing of microbiome samples collected 10 days post treatment showed similar overall microbial diversity in all six treatments, indicating a homogeneous complex microbial community (Fig. S6). Out of 50 predominant genus groups for each treatment (Fig. S7), 18 top genus groups were further analyzed (Fig. 5A).

In the rhizosphere soil with only sucrose addition, the relative abundance of *Bacillus* reached ~10.1% (CS1-3, Fig. 5A, B), while this ratio was only ~0.1% without sucrose addition (C1-3), indicating a 100-fold increase in the abundance of the native *Bacillus* species in the rhizosphere stimulated by sucrose. Further inoculation of WT *B. subtilis* caused no additive effect on *Bacillus* abundance (~9.6%) (AS1-3, Fig. 5A, B), possibly due to the upper limit of *Bacillus* enrichment in the tomato rhizosphere. Interestingly, when the Δ*sacB* cells were inoculated together with sucrose addition, the abundance of *Bacillus* genus only reached 6.6%, which was likely contributed by the native *Bacillus* genus in the soil (BS1-3, Fig. 5A, B). The relative abundance of *Bacillus* genus was about 0.28% when the Δ*sacB* cells were inoculated without sucrose (B1-3, Fig. 5A, B). In summary, our results clearly demonstrated the strong positive influence of sucrose on the increased prevalence of *Bacillus* genus in the tomato root rhizosphere. We were somewhat surprised that the initial inoculation of either the wild-type *B. subtilis* or the Δ*sacB* cells did not further increase the overall abundance of *Bacillus* in the rhizomicrobiome. In addition to strong positive influence of sucrose on the prevalence of *Bacillus* genus, the relative low abundance of *Pseudomonas* (0.05%, C1-3, Fig. 5B) was also elevated to 2.7% when inoculated with the wild type *B. subtilis* cells (A1-3, Fig. 5B) and further increased to 12.5% with simultaneous *B. subtilis* inoculation and sucrose supplementation (AS1-3, Fig. 5B). Addition of sucrose alone also elevated the abundance of *Pseudomonas*, although to a much lesser degree (from 0.05% in C1-3 to 0.27% in CS1-3, Fig. 5B).

Finally, we investigated if addition of sucrose may enhance biological control efficacy by promoting the root colonization and activities of beneficial bacteria such as *B. subtilis*. Tomato plants were challenged by two distinct pathogenic fungi, *Fusarium oxysporum* and *Botrytis cinereal*, respectively. Biological control experiments were carried out as described in the methods and disease index quantified. Our results showed that supplementation of sucrose to the 3610 formula both improved the suppression efficiency against the soil-borne disease caused by *Fusarium oxysporum* sp. *Lycopersici* (Fusarium wilt) by ~51%, and enhanced the resistance to the air-borne disease caused by *Botrytis cinerea* in tomato (gray mold) by about ~80% compared to no sucrose supplementation formula (Fig. 5C). Our results thus suggest that the direct and indirect effects by plant-released sucrose is likely more profound than simply being a carbon source for bacteria in the rhizosphere.

### Levan also induces SSM and hyper-flagellation in other soil bacteria

Levan was first found in the natto, a traditional Japanese health food fermented by *B. subtilis*, and was termed as “lävulan” by Lippmann in 1881 and denominated by Greig-Smith in 1901 [53, 54]. In addition to *B. subtilis*, a batch of other bacteria, including the genera of *Acetobacter*, *Erwinia*, *Gluconobacter*, *Halomonas*, *Microbacterium*, *Pseudomonas*, *Streptococcus*, and *Zymomonas*, have the *sacB* gene homologs, which share a common ancestor [44] (Fig. S8). It is possible that some of these *sacB*-containing bacterial species are also capable of producing levan when associated with plants. We thus wondered if levan could induce SSM in other soil bacteria as well. Our results showed that levan (0.2 g/L) can induce SSM and hyper-flagellation in selected rhizospheric bacteria such as *Serratia marcescens*, *Pectobacterium carotovorum*, *Xanthomonas oryzae*, *Pseudomonas protegens*, *etc* (Figs. 3E, S9). Again, this inducing effect was specific to levan since inulin, FOS, and dextran did not show any inducing effect (Fig. S9).

## Discussion

Rhizobacteria plays a pivotal role in protecting plants and promoting plant growth and health [2, 10, 17, 55]. How the beneficial bacteria recognize the signals from the plant host, colonize the roots, and ultimately establish an intimate relationship with the plant has been an intensely investigated topic in the field [5, 6, 20, 56–58]. One of the specific focuses is on plant root-released nutrients and their impact on the rhizospheric bacteria [8, 14, 16, 32, 59]. Among those root-released nutrients, sucrose is found most abundantly released into the rhizosphere [32, 60, 61]. Sucrose is uniquely important to the plants since photosynthetic plants primarily use sucrose as a fixed carbon transport and storage mechanism among different tissues [60]. To the bacteria, however, sucrose is often regarded as a common carbon source among many other nutrients released by the roots. Here, we presented evidence for a more comprehensive and unique role of the root-released sucrose in inducing root colonization and promoting rhizosphere prevalence by *B. subtilis*. Our study also suggests that sucrose may be important as well to those rhizospheric bacteria possessing the *sacB* pathway. Sucrose was found to be the most abundant sugar in the soil near the root tip of the annual grass *Avena barbata* [61]. Sucrose secretion by the plant hosts was shown to be significantly enhanced during various biotic and abiotic attacks [62, 63]. It is plausible that plants may have employed root secretion of sucrose as a general defense mechanism during stress response by selectively enhancing root colonization of sucrose-responsive beneficial bacteria such as *B. subtilis*. On the other hand, rhizospheric bacteria like *Bacillus* may have evolved to use sucrose as a unique plant signal that promotes root colonization [64].

Chemotaxis is proposed to play a key role in bacterial root colonization [56]. In a previous study, application of various mutants of *B. subtilis* provided convincing evidence that the function of motility and chemotaxis is key to bacterial root colonization [20]. However, since chemotaxis is perceived as a directed swimming driven by flagella in aqueous environments [65], swimming-driven motility may not fully explain bacteria swiftly migrate in the rhizosphere, considering the semiarid or arid nature of the rhizosphere soil. It is quite possible that soil bacteria oftentimes have to reply on certain types of solid or semisolid surface motility in order to efficiently perform rhizosphere migration. In this study, we characterized solid-surface migration by *B. subtilis* stimulated by sucrose. Sucrose acts as a signal to initiate a signaling cascade leading to biosynthesis of levan and strong production of surfactin. Surfactin may have multiple roles in promoting *B. subtilis* rhizosphere colonization. One, it functions as a biosurfactant to promote surface migration; second, it acts as a signal to stimulate hyper-flagellation of *B. subtilis* cells through a yet unknown mechanism; third, surfactin may promote root-associated biofilm formation by *B. subtilis*;[64] fourth, surfactin, as an antimicrobial peptide [36], may help *B. subtilis* compete with other species in the rhizosphere. Thus, root-released sucrose and the signaling cascade are indeed critical to promote rhizosphere colonization and competition by *B. subtilis* and other *Bacillus* species. A previously published study suggested that surfactin is not important in root colonization by *B. subtilis* [66]. We hope to point out that root colonization assays in that study were primarily performed in aqueous media with *Arabidopsis* roots and the bacterial root colonization was measured by CFU counting within 24–48 h after *B. subtilis* inoculation (based on descriptions in the method) [66]. Surfactin is not known to be important in swimming motility and chemotaxis in aqueous environments. In this study, root colonization assays were performed exclusively in pot soil on tomato plant. Therefore, results from both studies could be potentially valid because of very different experimental settings used in root colonization assays. Even so, in our study, in the absence of added sucrose, the surfactin mutant only showed an about 1.8-fold reduction in root colonization compared to the wild type (Fig. S5). We speculate that this could be in part due to initial bacterial inoculation and periodical watering during plant growth. These watering events may allow surfactin mutants to perform swimming motility and root colonization to some degree. In other words, the importance of surfactin on *B. subtilis* root colonization could depend on the environment; a more aqueous environment may diminish the importance of surfactin in root colonization.

In this study, we also revisited the role of levan, identified a century ago and nowadays widely applied in the industries of food, cosmic, and health care [44]. The biological function of levan and the metabolic benefit of such a pathway to the producing bacteria are not clear. Our study suggests that levan acts as one of the chemical signals in the signaling cascade that leads to effective root colonization by *B. subtilis*. Levan was recently found in the biofilm of *B. subtilis* [67], which is known to play an important role in establishing symbiotic interactions between *B. subtilis* and the plant. In another published study [68], it was reported that levan can stimulate soil aggregation, which in turn could significantly influence spreading and rhizosphere presence by the soil bacteria. Homologs of the *sacB* gene are found in a number of rhizospheric bacteria, and more interestingly, levan can stimulate similar solid-surface movement and hyper-flagellation in other rhizospheric bacteria (Figs. S6, S7). Taken together, these results strongly suggest that the *sacB* pathway and levan could play an important, previously less characterized, role in root colonization in other soil bacteria as well. Interestingly, sucrose also showed a mild effect on the root colonization by both the flagellar and surfactin mutants (Fig. S5). We speculate that this could be due to the effect of levan, the polysaccharide made from sucrose, which itself is proposed to have biosurfactant-like activities. It is possible that sucrose may stimulate root colonization through other unknown mechanisms. For example, sucrose may alter metabolic status of the cells (not necessarily the growth rate), which could stimulate root colonization independent on enhancing surfactin production.

Finally, our findings suggest a practicable approach to boost colonization of beneficial *Bacillus* species and intervene rhizosphere microbiome that promotes the disease control efficacy against some soil-borne phytopathogens [69, 70]. Application of sucrose may significantly enhance the biological control efficacy of BCAs, which now demonstrate a rising popularity in the agricultural field due to their biosafety and environment friendliness compared to traditional chemical pesticides and fertilizers. The increased disease control efficacy may be due to competitive root colonization, and production of surfactin and other antibiotic compounds by beneficial bacteria such as *Bacillus* and *Pseudomonas* species, as discussed in a number of previously published studies [2, 27, 36, 71].

## Methods

### Strains and growth media

Bacterial strains including *Bacillu*s *subtilis*, their isogenic derivatives, and various other bacterial strains (Table S1) used in this study were routinely grown in Lysogeny Broth (LB). The recipe for the minimal medium MSgg was described previously, in which 0.5% (w/v) glycerol is supplied as the carbon source [34]. M9 minimal medium [35] used in this study contained 0.4% (w/v) glucose as the carbon source prior to supplementation of any other sugars. For assaying SSM, different sugars were supplemented to the solid LB media at the concentration of 5 g/L unless specifically indicated. When required, antibiotics were supplemented at the final concentrations as follows for *B. subtilis*: erythromycin 1 µg/mL, tetracycline 5 µg/mL, chloramphenicol 20 µg/mL, spectinomycin 100 µg/mL, and kanamycin 10 µg/mL. The pathogenic fungal strains were grown on Potato Dextrose Agar (PDA) media at 25 °C [72]. Sugars were purchased from Thermo Fisher Scientific (Burlington, MA, USA). Chemicals including surfactin, levan, inulin, FOS, and dextran were purchased from Sigma-Aldrich (St. Louis, MO, USA). Levanbiose was purchased from Megazyme (Megazyme International Ltd., Ireland). Restriction enzymes were purchased from New England Biolabs (Ipswitch, MA, USA).

### Strain construction

The general methods for molecular cloning followed published protocols [35]. Preparation of genomic DNAs from *B. subtilis* was described previously [73]. Restriction enzymes were used according to the manufacturer’s instructions. To construct the insertional deletion mutants in the *B. subtilis* NCIB 3610 (hereafter abbreviated as 3610), the genomic DNA of the corresponding mutant of the *B. subtilis* 168 strain (obtained from the Bacillus Genetic Stock Center, BGSC) which harbors an antibiotic resistance gene cassette-tagged insertional deletion mutation in the target gene, was prepared and introduced into the *B. subtilis* 3610 by genetic transformation as previously described [74]. The insertional deletion was verified by PCR amplification of the genomic region and DNA sequencing of the amplified DNA fragment. To construct various reporter strains in the 3610 background, the genomic DNA containing the reporter construct and antibiotic resistance marker were prepared from previously constructed strains (Table S1) and introduced into 3610 or its derivatives by genetic transformation.

### Tomato root colonization

The tomato cultivar “Zhongza 9” was procured from the China Vegetable Seed Technology Co., LTD (Beijing, China). The tomato seeds were surface sterilized by a 60 s soak in 75% (v/v) ethanol, followed by 5 min in sodium hypochloride solution (10% active chlorine), and by three subsequent wash steps with sterile water for 10 min each. Sanitized tomato seeds were deposited into the petri dish (10 cm in diameter), which was covered by a sterilized filter paper and contained 1 mL of sterilized water. The petri dish was incubated in moisture chambers at 28 °C for 48 h. When seeds started to germinate, the seeds were transplanted to plastic pots (70 mm × 70 mm × 75 mm) containing 15 g sterilized vermiculites, and the pots were incubated in moisture chambers (28 °C/20 °C day/night temperatures, 3500 Lux light for 16 h/d, and 70% relative humidity). The pots were watered weekly with nutrient solution. After 15 days of incubation, 5 mL cell suspension containing freshly cultivated *B. subtilis* cells (1.0 × 10^8^ CFU/mL) and supplemented with different sugars (5 g/L) was added to the pot by pouring it to the root surrounding. After another 3 days of incubation, roots of seedling were taken out and rinsed with sterilized water; 1 cm root ripening zone for each sample was then collected and quickly stored in the sterile Eppendorf tube for LSCM and plate recovery counting.

### *Arabidopsis thaliana* root colonization

Seeds of the *A. thaliana* mutants Δ*STP1* (SALK-048848C), Δ*AtSUC2* (SALK-038124), and Δ*AtSUC3* (SALK-077723) were obtained from Arabidopsis Biological Resource Center (The Ohio State University, USA). The wild-type *A. thaliana* used in this study is Col-0 (a kind gift from Dr. Pengmin Zhou, China Agricultural University, Beijing, China). The *A. thaliana* seeds were surface sterilized by a 30 s soak in 75% (v/v) ethanol, followed by 5 min in sodium hypochloride (10% active chlorine) and by three subsequent wash steps with sterile water for at least 10 min each. Sterilized seeds were then transferred onto the 0.7% Murashige and Skoog agar plate and cold-incubated at 4 °C and dark for 3 days for optimal germination. The plates were then incubated in moisture chambers at 22 °C for 3 days until the length of the *A. thaliana* roots reached about 1 cm. The seedlings were transplanted to the plastic pots (70 mm × 70 mm × 75 mm) containing 15 g sterilized vermiculites, with an initial moisture content of 100% (v/w) and incubated in moisture chambers (22 °C/22 °C day/night temperatures, 3000 Lux light for 16 h/d, and 60% relative humidity), and watered weekly with nutrient solution. After 20 days of incubation, 5 mL overnight cultivated cell suspension of *B. subtilis* (1.0 × 10^8^ CFU/mL), supplemented with different sugars (5 g/L), was inoculated by pouring to the root. After another 3 days of incubation, roots of seedlings were taken out and rinsed with sterilized water; then 1 cm root ripening zone for each sample was taken and quickly stored in the sterile Eppendorf tube for Laser confocal microscopic imaging and cell recovery counting.

### Cell recovery counting

One milliliter of sterile water was added to the Eppendorf tube containing 1 cm preprocessed root and vortexed for 10 min. The suspension was serially diluted with distilled water. 100 μL cell suspension from various dilutions (10^−2^, 10^–3^, or 10^−4^) was plated onto LB plates supplemented with the appropriate antibiotic. Plates were incubated overnight at 35 °C. The number of colony forming unites (CFU) per mm root was determined. The experiment was repeated three times with ten root samples per replicate.

### Biological control

Seeds of the tomato cultivar “Zhongza 9” were pre-germinated as described above, and each germinated seedling was sowed into a plug of the nursery tray that contained dry sterilized artificial mixed soil (vermiculite: turf: soil = 1:1:1, v/v). After 20 d of cultivation, seedlings were transplanted into plastic pots (70 mm × 70 mm × 75 mm) that contained 100 g sterilized artificial soil. The biocontrol experiments were conducted in triplicate (20 seedlings for each treatment) in an artificial climate chambers (25 °C/18 °C day/night temperatures, 3500 Lux light for 16 h/d, and 80% relative humidity).

For the biocontrol assay of root rot disease, 20 mL cell suspension (1.0 × 10^8^ cfu ml^−1^) of *B. subtilis* 3610 or its derivatives, with or without 0.5% (w/v) sucrose, was poured into each pot. 6 h later, 20 mL conidial suspension (5.0 × 10^6^ cfu mL^−1^) of *Fusarium oxysporum* f. sp. *Lycopersici* was poured into each pot. The disease control efficacy was surveyed and calculated according to a published protocol [75]. For the biocontrol assay of gray mold disease, 48 h after tomato seedling transplantation and treatment by *B. subtilis* cell suspension, 2 mL conidial suspension of *Botrytis cinereal* (1.0 × 10^5^ cfu mL^−1^) was sprayed to every seedling. The disease control efficacy was surveyed and calculated accordingly [76].

### Solid surface motility (SSM)

Cells were grown overnight at 37 °C in LB broth. Solid LB plates (1.5% agar, w/v) supplemented with sugars were freshly prepared and dried for 30 min in a laminar flow hood prior to use. 2 µL cell suspension was inoculated onto the plate, dried for 5 min on bench, and incubated at 37 °C for 8–12 h with the plates facing upside down. Each data point represents an average from three independent experiments. Flagellum staining of the cells collected from the plates for SSM was performed as described previously [77].

To determine the influence of exogenous chemical compounds on SSM by *B. subtilis*, pure chemicals were supplemented to the SLB plates as indicated. Surfactin was dissolved in 100% methanol at the stock concentration of 180 mg/mL. 10 µL of the surfactin stock solution was added into an oxford cup 1 cm away from the center of the plate. Other chemical compounds were dissolved in sterile water at the concentration of 20 mg/mL. Then, 100 µL stock solution with the indicated concentration was added into LB medium and dried for 20 min in a laminar flow hood.

### Soil collection and rhizosphere sampling

Soil used in plant root microbiome studies were collected from a wheat and maize rotation field in Wen’an County (116.66°E and 38.93°N), Hebei Province, China. The field soil was collected on June 14, 2019, prior to the wheat harvest and maize seeding when the diversity and richness of the soil microbial community was high. Tomato cultivar (Zhongza 9) was grown in this natural soil and inoculated with the wild type or the *sacB* deletion mutant of *B. subtilis* 3610 and supplemented with or without sucrose. Tomato seedlings were grown in square PVC pots (70 mm × 70 mm × 75 mm) containing 100 g soil, with an initial moisture content of 30% (v/w). During the 25-day period, tomatoes were grown in the moisture chamber as described above. Subsequently, the experimental treatments contained: (1) inoculation of WT *B. subtilis* or (2) Δ*sacB* mutant without sugar supplementation; (3) inoculation of WT *B. subtilis* or (4) Δ*sacB* mutant with sucrose supplementation; (5) with sucrose supplementation but no bacteria; (6) without sucrose supplementation and no bacteria as a negative control. For the treatments, overnight cultivated bacterial suspensions (1.0 × 10^8^ cells/mL, 5 mL per pot) and/or 73 mM sucrose (10 mL per pot) were dripped slowly into the soil surrounding the plant root, and water (15 mL per pot) as the negative control. For all treatments, the experiment was performed in three biological replicates (10 tomato plants per replicate). After another 10 days of incubation, rhizosphere soil samples were obtained. To do so, the plant was uprooted by scoop and loosely adhering soil was removed by shaking vigorously. The tightly adhering soil around the root, regarded as rhizosphere soil, was placed in a sterilized 50 mL Falcon tube and visible plant roots were removed carefully from each sample. Rhizosphere samples were stored at −80 °C for further use.

### Statistical analyses

Statistical analysis was performed using SPSS software version 20.0 (SPSS Inc., Chicago, IL, USA). Comparisons were done using one-way analysis of variance, followed by Tukey post-tests (set at 5%) which correct for multiple comparisons using statistical hypothesis testing. Means of different treatments were compared using the least significant difference (LSD) at a 0.05 (*) or 0.01 (**) level of probability. The letters above the columns indicate statistically significant differences based on Student’s *t* test (the capital letters: *p* < 0.01; the lower letters: *P* < 0.05). The Pearson correlation coefficient was analyzed using double-variable analysis. All error bars show the mean and standard deviation (SD). Each experimental treatment had three biological replicates.

The following assays were described in Supplementary Methods:

1. 16S rRNA gene MiSeq sequencing and sequencing analyses.
2. Microscopic analysis, including laser scanning confocal microscopy, light microscopy, and photography.
3. Assays of surfactin production by HPLC.
4. Assays of β-galactosidase activities.
5. Quantification of cell fluorescence intensity.

## Supplementary information

Supplementary materials
