# Supplementary material for: Sucrose triggers a novel signaling cascade promoting Bacillus subtilis rhizosphere colonization
Source: ISME J. 2021 Mar 26;15(9):2723–37. doi: 10.1038/s41396-021-00966-2 (PMC8397739; doi:10.1038/s41396-021-00966-2)
Supplement: Supplementary file 1 — Supplementary materials [file 41396_2021_966_MOESM1_ESM.pdf]

Supplementary Materials_Tian et al.

Supplemental Methods.

16S rRNA gene MiSeq sequencing.

The genomic DNA of the microbial community in the rhizosphere samples was extracted using the Fast DNA® Spin Kit for Soil (MP Biomedicals, U.S.A) according to manufacturer's instructions. The DNA extract was checked on 1% agarose gel. The concentration and purity of the extracted genomic DNA were determined with ScanDrop 100 spectrophotometer (Analytic Jena AG, Germany). The genomic DNA extracts were stored at -20˚C for further use.

16S rRNA gene amplification and sequencing were conducted by Majorbio Bio-Pharm Technology Co., Ltd. (Shanghai, China). Briefly, the V3-V4 hypervariable region of the 16S rRNA gene was amplified by PCR using primers 338F (5'-ACTCCTACGGGAGGCAGCAG-3') and 806R (5'-GGACTACHVGGGTWTCTAAT-3') in an ABIGeneAmp®9700 PCR thermocycler (ABI, CA, USA). The PCR amplification of 16S rRNA gene was performed as follows: initial denaturation at 95 °C for 3 min, followed by 27 cycles of denaturing at 95 °C for 30 s, annealing at 55 °C for 30 s and extension at 72 °C for 45 s, and single extension at 72 °C for 10 min, and terminate at 4 °C. The PCR mixtures contain 5 × *TransStart* FastPfu buffer 4 μL, 2.5 mM dNTPs 2 μL, forward primer (5 μM) 0.8 μL, reverse primer (5 μM) 0.8 μL, *TransStart* FastPfu DNA Polymerase 0.4 μL, template DNA 10 ng, and finally ddH_2O up to 20 μL. PCR reactions were performed in triplicate. The PCR product was extracted from 2% agarose gel, purified using the AxyPrep DNA Gel Extraction Kit (Axygen Biosciences, Union City, CA, USA) according to manufacturer's instructions and quantified using Quantus™ Fluorometer (Promega, USA). Purified amplicons were pooled in equimolar and paired-end

sequenced (2 × 300 bp) on an Illumina MiSeq platform (Illumina, San Diego, USA) according to the standard protocols by Majorbio Bio-Pharm Technology Co. Ltd. (Shanghai, China). All of the raw reads are archived at the NCBI Sequence Read Archive (SRA) database (accession number: SRR 10279951- 10279968).

Sequencing analyses.

The raw 16S rRNA gene sequencing reads were demultiplexed, quality-filtered by Trimmomatic (version 0.39, http:// usadellab.org/cms/) and merged by FLASH with the following criteria: (i) the 300 bp reads were truncated at any site receiving an average quality score of <20 over a 50 bp sliding window, and the truncated reads shorter than 50 bp were discarded; reads containing ambiguous characters were also discarded; (ii) only overlapping sequences longer than 10 bp were assembled according to their overlapped sequences. The maximum mismatch ratio of overlap region is 0.2. Reads that could not be assembled were discarded; (iii) Samples were distinguished according to the barcode and primers, and the sequence direction was adjusted based on exact barcode matching (allowing 2 nucleotide mismatch in primer matching). Operational taxonomic units (OTUs) with 97% similarity cutoff were clustered using UPARSE (version7.1, http://drive5.com/uparse/), and chimeric sequences were identified and removed. The taxonomy of each OTU representative sequence was analyzed by RDP Classifier (http://rdp.cme.msu.edu/) against the 16S rRNA database (SILVA SSU128) using confidence threshold of 70%.

Quantification of the fluorescent signal density.

For imaging of the P_{srfAA}-*gfp* fluorescent reporter strain (CY106), cells were grown in LB broth to log phase. 2 μL log phase culture was spotted onto the center of the solid

LB plates supplemented with or without sucrose (5 g/L) and plates were incubated at 37C. After 4 hours of incubation, cells were collected from the plates, spun down, washed with PBS buffer once, and resuspended in 100 μL of PBS buffer. 2 μL of the resuspension was placed on a 1% (w/v) agarose pad, covered with a cover slip, and observed under fluorescent microscopy. Imaging of different samples was conducted using the same exposure settings. To quantify the fluorescence intensity of the P_{srfAA}-*gfp* expressing cells fluorescence of a total of about 200 cells was quantified using the MicrobeJ plugin for ImageJ [1,2]. For integrated intensity, areas of the selected cell and background fluorescence were measured, total cell fluorescence of each cell was calculated by using integrated intensity to subtract mean background fluorescence.

Assays of β-galactosidase activities.

To measure β-galactosidase activity, the reporter strain (KG203) was inoculated on solid LB plates (1.5% agar, w/v) supplemented either sucrose or glucose (5 g/L) or no sugar addition as described in the solid surface motility assay. After 4 h of cultivation, cells were collected from the edge of the colonies on the plates, and β-galactosidase specific activities were quantified as described [3].

Laser scanning confocal microscopy (LSCM)

Collected root samples were mounted on microscope slides (VWR, USA) and were directly observed under the laser confocal microscope (Zeiss LSM 800) at the excitation wavelength of 561 nm. All images were taken at the same exposure time and processed identically. The images were acquired using ZEN 2.3 (blue edition) and exported as tiff files. Each image is a representative of at least 10 root colonization assays performed in three independent experiments.

Light microscopy and photography

For phase or regular imaging, three different devices were used: a Nikon SMZ800N stereomicroscope was used to capture colony morphology; photographs for cell motility on agar plates were recorded by a canon EOS 750D digital camera; all high-magnification imaging at the cellular level was done with the Leica DM3000 workstation (2000× magnification).

Surfactin production assay.

For analysis of surfactin production, cells were incubated on either solid LB plates or solid MSgg[4] media with or without various sugars at 30°C for 12 h or 72 h, respectively, and samples of colony were taken by a hole puncher with a diameter of 0.5 cm. 10 pieces of samples from each treatment were put into flaks with 50 mL ddH_2O and shaken for 5 min for surfactin extraction. 45 mL liquid was taken and centrifuged (5 min, 14,200g) to remove cells, and the supernatant was filter-sterilized. 40 mL cell free culture filtrate was then mixed with 40 mL acetonitrile (Merck, PA, USA). Crude surfactin was extracted, further purified by an adapted SPE (solid phase extraction) technique, and detected by the Accela HPLC system (Thermo Fisher Scientific) as previously described [5].

Supplemental Figure Legends.

Figure S1. The growth profile of *B. subtilis* 3610 and the mutants (Δ*sacA* and Δ*sacB*) in the minimal MSgg, or M9, or LB media supplemented with various sugars. MSgg contains 0.5% glycerol as the carbon source. For M9, 0.4% (w/v) glucose was added as the carbon source prior to addition of any other sugars. For supplementation of different sugars (Suc: sucrose, Glu: glucose, Fru: fructose, Mal: maltose, Inu: inulin, FOS: fructooligosaccharide, Dex: dextran or Lev: levan), the indicated sugar was added into the above media at the final concentration of 5 g/L. Glu/Fru in (a) indicates a combination of both glucose and fructose, each at 2.5 g/L. The growth profile of *B. subtilis* 3610 and the Δ*sacA* and Δ*sacB* mutants were similarly assayed in MSgg (d), M9 (e), and LB (f) with or without addition of sucrose (Suc, 5 g/L). The data represents the mean of three independent assays performed in duplicate. Error bars indicate standard deviations.

Figure S2. Effects of various supplemented sugars on *B. subtilis* colony morphology and solid surface motility (SSM). a. SSM by the WT and the Δ*hag* mutant on solid LB media (1.5% agar, w/v) with or without supplementation of 5 g/L sucrose. **b.** Sucrose specifically triggers a robust SSM of 3610 on solid LB media (1.5% agar, w/v). Top-down view of colony morphology in which *B. subtilis* 3610 was incubated for 24 h at 30 °C in the presence of different sugars as indicated (5 g/L). **c.** Levan specifically induces SSM by *B. subtilis* 3610. 3610 was incubated for 16 h on solid LB plates supplemented with levan or various plant polysaccharides (Lev: levan, Xyl: xylan, Pec: pectin, Cel: cellulose) at 0.2 g/L. Pictures are representatives of at least 3 independent plates. Results are representatives of three experiments.

Figure S3. The phylogenetic analysis of 16S rRNA gene sequences of the *Bacillus* strains. Sequences of *Bacillus* strains (3610, NCD-2, 9407, TB1501, TB1340 and B579) were downloaded from Genebank database (www.ncbi.nlm.nih.gov). The tree was constructed in MEGA 7.0 using the Neighbor-Joining method, with 1,000 bootstrap samplings.

Figure S4. Sucrose-induced surfactin production depends on *sacB*, but not *sacA*. The effect of sucrose-induced surfactin yield (μg/cm^2) was assayed on solid LB (**a,** incubation for 12 h) agar plates and MSgg (**b,** incubation for 30 h) agar plates (1.5% agar, w/v) in the presence or absence of sucrose (5 g/L) in the WT (3610), Δ*sacA* (Tm13) and Δ*sacB* (Tm14) mutants. Samples were collected from the plates. Surfactin was extracted, and the amount of surfactin was determined by HPLC as described in the method. The error bars represent standard deviations from triplicate assays. ** indicates p value <0.01; NS, no statistical difference.

Figure S5. Δ*hag* and Δ*srfAA* deletion mutants of *B. subtilis* showed decreased root colonization. The difference on the tomato root colonization between the WT and the Δ*hag* (Tm05) and Δ*srfAA* (Tm01) deletion mutants was determined by counting colony forming unit (CFU) per mm root length by plate recovery counting as described in the method. The data represents the mean of three independent assays performed in duplicate. Error bar indicates standard deviations, and single asterisks or double asterisks (∗ or ∗∗) indicate significant differences by LSD at $p < 0.05$ or $p < 0.01$. S stands for supplementation of sucrose.

Figure S6. Quantification of 16S rRNA gene copies in the rhizomicrobiome

samples. The quantitative bacterial population (16S rRNA gene copies/gram soil) of the tomato root rhizosphere inoculated with *B. subtilis* 3610 or the Δ*sacB* mutant and with or without supplementation of sucrose. A: *B. subtilis* 3610, AS: *B. subtilis* 3610 plus sucrose, B: Δ*sacB* mutant, BS: Δ*sacB* mutant plus sucrose, C: no 3610 and sucrose, CS: with only sucrose. The letters above the columns indicate statistically significant differences based on Student's t-test ($p < 0.05$). Values are given as mean of three independent biological replicates and the bars represent standard deviations.

Figure S7. The relative abundance of 50 top genus groups by community barplot analysis. 51 different colors represent different bacterial genus.

Figure S8. The phylogenetic tree based on putative SacB protein homologs in various bacteria. These SacB protein sequences of the related strains were downloaded from UniProt database (www.uniprot.org). The blue branches show the position of SacB in *Bacillus*. The tree was constructed using ClustalW in MEGA 7.0, with 1,000 bootstrap samplings. Bar represents 0.4 substitutions per amino acid position.

Figure S9. Levan induces SSM in other soil bacteria. Soil bacteria (*Serratia marcescens* T4-3, *Pectobacterium carotovorum* subsp. carotovorum Z3-3, *Xanthomonas oryzae* pv. oryzae PXO99F, *Pseudmonas. protegens* pf-5, and *P. fluorescens* 2p24) were incubated for 16 h on solid LB plates supplemented with levan or its structural analogues (inulin, FOS, and dextran) at the concentration of 0.2 g/L. Pictures are representatives of at least 3 independent experiments.

Table S1. Strains used in this study

**Strains**	**Relevant phenotype**	**Reference**
***Bacillus.* sp**		
NCIB3610	wild-type *B. subtilis* capable of robust biofilms	[4]
168	domesticated *B. subtilis* strain	[6]
BKK03480	168 *srfAA::kan*	BGSC
BKK38040	168 *sacA::kan*	BGSC
BKE34450	168 *sacB::erm*	BGSC
BKE34460	168 *levB::erm*	BGSC
BKK27030	168 *sacC::kan*	BGSC
BKK34470	168 *yveA::kan*	BGSC
NCD-2	wild-type *B. subtilis*	[7]
B579	wild-type *B. subtilis*	[8]
CAU9407	wild-type *B. subtilis*	[9]
TB1340	wild-type *B. velezensis*	CP022531.1*
TB1501	wild-type *B. velezensis*	MT946907*
B905	wild-type *B. cereus*	[10]
TG071	*B. cereus* B905 harboring pGFP78, Tet^{R}	[10]
CY106	3610 *lacA*::P_{srfAA}-*gfp*, mls^{R} , DL746→3610	this study
EH039	3610 *amyE*::P_{comGA}-*gfp*, Cm^{R}	this study
KG203	3610 *amyE*::P_{srfAA}-*lac*Z, cm^{R}	this study
Tm01	3610 *srfAA*::*kan*	this study
Tm02	NCD-2 *srfAA*::*kan*	gift from Ping Ma
Tm03	9407 *srfAA*::*kan*	[9]
Tm05	3610 *hag*::*mls*	this study
Tm13	3610 *sacA*::*kan*, BKK38040→3610	this study
Tm14	3610 *sacB*::*kan*, BKK34450→3610	this study
Tm23	3610 *sacC*::*kan*, BKK27030→3610	this study
Tm26	3610 *levB*::*erm*, BKE34460→3610	this study
Tm28	3610 *yveA*::*kan*, BKK34470→3610	this study
Tm31	3610 *sacA*::*kan*, *amyE*::$P_{hyspank}$-*mKate2*::*chl*	this study
Tm32	3610 *sacB*::*erm, amyE*::$P_{hyspank}$-*mKate2*::*chl*	this study
YC843	3610 *amyE*::$P_{hyspank}$-*mKate2*::*chl*	this study
**Others**		
T4-3	wild-type *Serratia marcescens*	laboratory collection
Z3-3	wild-type *Pectobacterium carotovorum* subsp. carotovorum	laboratory collection
PXO99F	wild-type *Xanthomonas oryzae* pv. oryzae	laboratory collection
AACOO1	wild-type *Acidovorax citrulli*	laboratory collection
PsmDG34	wild-type *Pseudomonas syringae* pv. maculicola	laboratory collection
pf-5	wild-type *P. protegens*	laboratory collection
2P24	wild-type *P. fluorescens*	laboratory collection
2-79	wild-type *P. fluorescens*	laboratory collection
PA01	wild-type *P. aeruginosa*	laboratory collection
T63	wild-type *Ochrobactrum sp.*	laboratory collection
	Fusarium oxysporum f. sp. *lycopersici*	laboratory collection
	Botrytis cinerea	laboratory collection

*GenBank access numbers

Supplemental references

1. Ducret, A., Quardokus, E. M. & Brun, Y. V. MicrobeJ, a tool for high throughput bacterial cell detection and quantitative analysis. *Nat Microbiol* **1**, 16077, doi:10.1038/nmicrobiol.2016.77 (2016).
2. Gozzi, K. *et al.* *Bacillus subtilis* utilizes the DNA damage response to manage multicellular development. *npj Biofilms Microbiomes* **3**, 8, doi:10.1038/s41522-017-0016-3 (2017).
3. She, Q. *et al.* Negative Interplay between Biofilm Formation and Competence in the Environmental Strains of *Bacillus subtilis*. *mSystems* **5** (2020).
4. Branda, S. S., González-Pastor, J. E., Ben-Yehuda, S., Losick, R. & Kolter, R. Fruiting body formation by *Bacillus subtilis*. *Proc Nat Acad Sci USA* **98**, 11621-11626 (2001).
5. Aleti, G. *et al.* Surfactin variants mediate species-specific biofilm formation and root colonization in *Bacillus*. *Environ Microbiol* **18**, 2634-2645 (2016).
6. Zeigler, D. R. *et al.* The Origins of 168, W23, and Other *Bacillus subtilis* Legacy Strains. *J Bacteriol* **190**, 6983-6995, doi:10.1128/jb.00722-08 (2008).
7. Guo, Q. *et al.* Fengycin produced by *Bacillus subtilis* NCD-2 plays a major role in biocontrol of cotton seedling damping-off disease. *Microbiol Res* **169**, 533-540, doi:https://doi.org/10.1016/j.micres.2013.12.001 (2014).
8. Chen, F. *et al.* Quantitative changes of plant defense enzymes and phytohormone in biocontrol of cucumber Fusarium wilt by *Bacillus subtilis* B579. *World J Microbiol Biotech* **26**, 675-684 (2010).
9. Fan, H. *et al.* Biocontrol of bacterial fruit blotch by *Bacillus subtilis* 9407 via surfactin-mediated antibacterial activity and colonization. *Front Microbiol* **8**, 1973-1988 (2017).
10. Gao, T. *et al.* The phosphotransferase system gene *ptsH* plays an important role in MnSOD production, biofilm formation, swarming motility, and root colonization in *Bacillus cereus* 905. *Res Microbiol* **170**, 86-96, doi:https://doi.org/10.1016/j.resmic.2018.10.002 (2019).

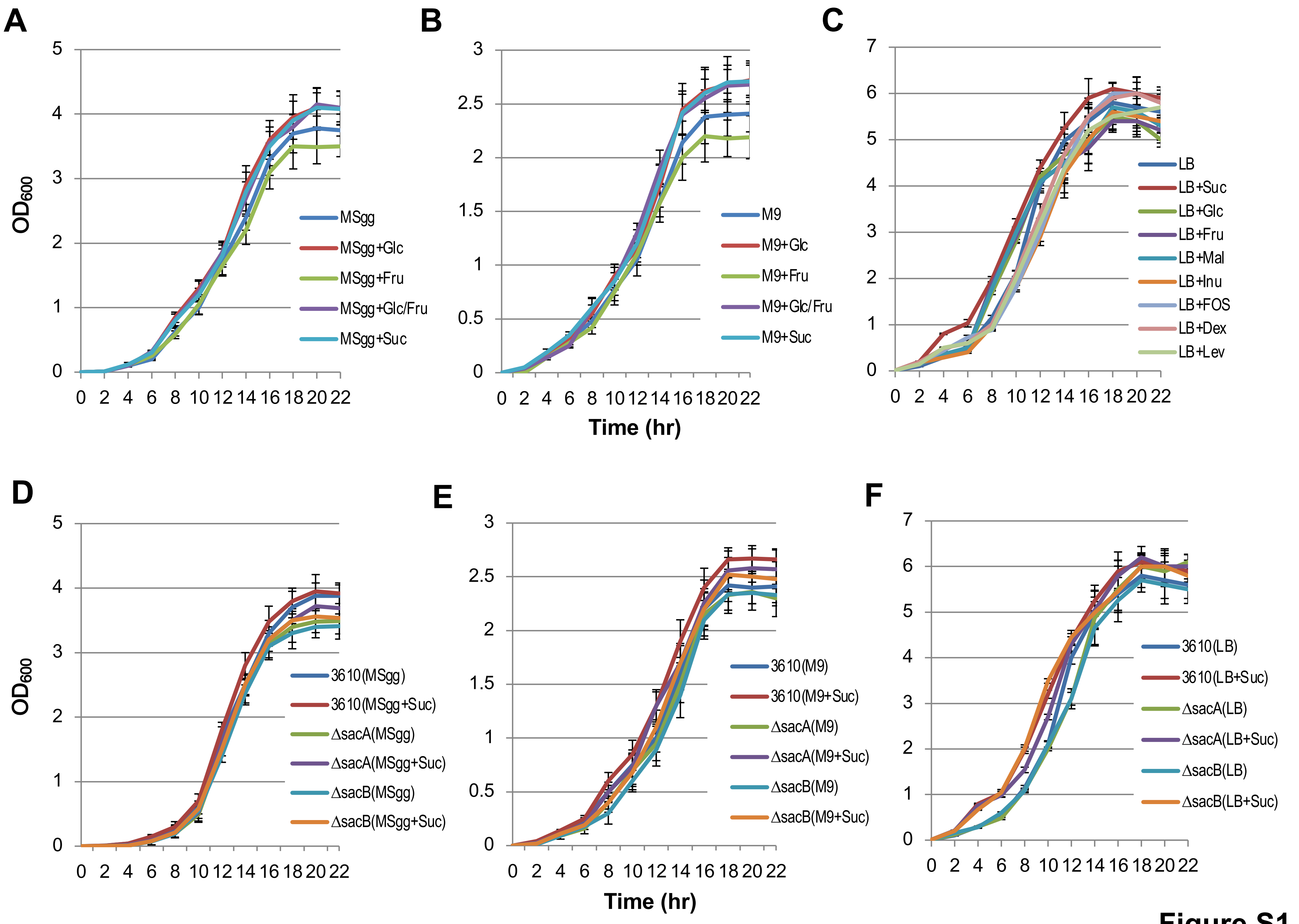

A
B
C
D
E
F
OD600
Time (hr)
MSgg
MSgg+Glc
MSgg+Fru
MSgg+Glc/Fru
MSgg+Suc
M9
M9+Glc
M9+Fru
M9+Glc/Fru
M9+Suc
LB
LB+Suc
LB+Glc
LB+Fru
LB+Mal
LB+Inu
LB+FOS
LB+Dex
LB+Lev
3610(MSgg)
3610(MSgg+Suc)
ΔsacA(MSgg)
ΔsacA(MSgg+Suc)
ΔsacB(MSgg)
ΔsacB(MSgg+Suc)
3610(M9)
3610(M9+Suc)
ΔsacA(M9)
ΔsacA(M9+Suc)
ΔsacB(M9)
ΔsacB(M9+Suc)
3610(LB)
3610(LB+Suc)
ΔsacA(LB)
ΔsacA(LB+Suc)
ΔsacB(LB)
ΔsacB(LB+Suc)

Figure S1

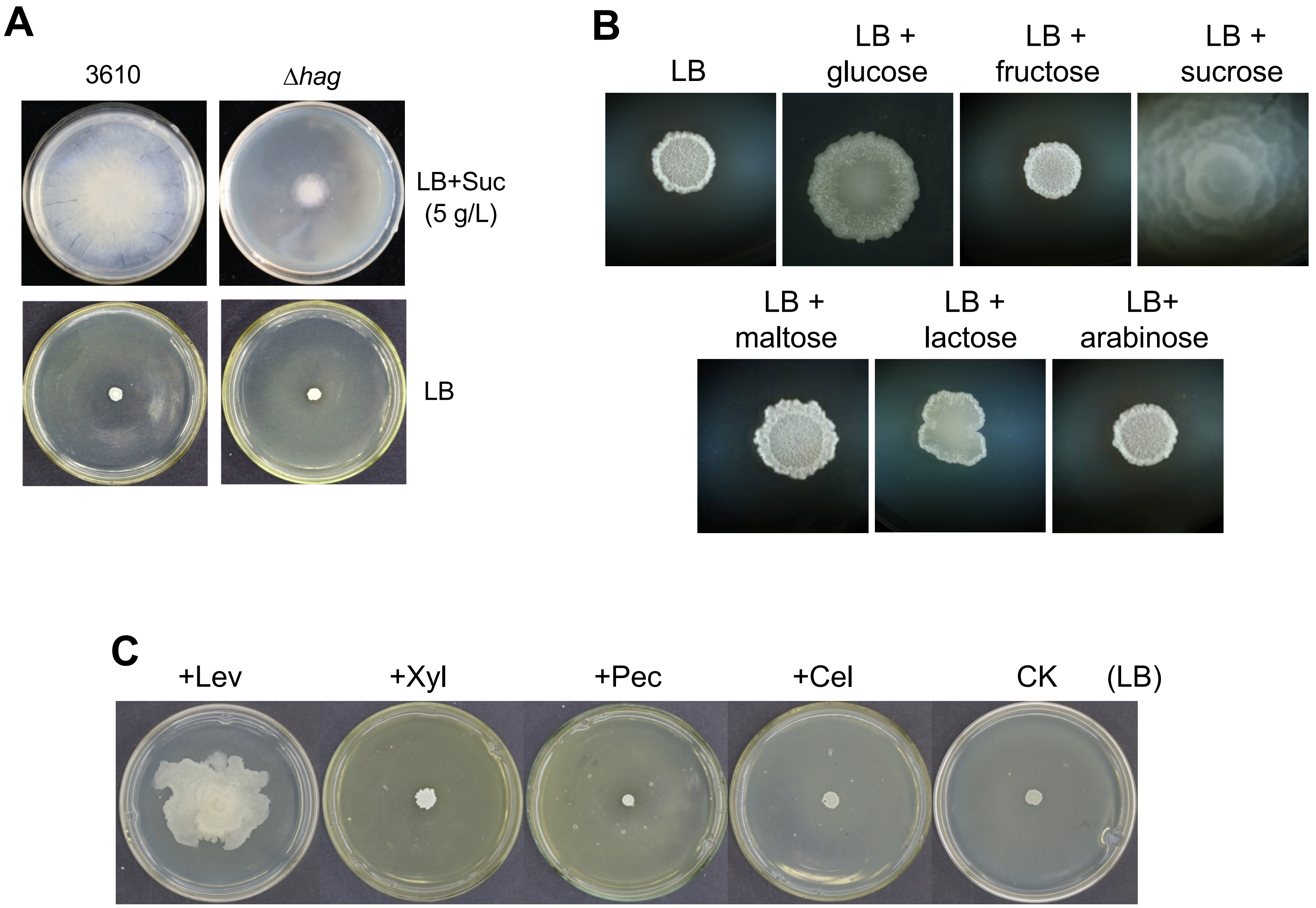
A
3610
Δhag
LB+Suc
(5 g/L)
LB
B
LB
LB +
glucose
LB +
fructose
LB +
sucrose
LB +
maltose
LB +
lactose
LB+
arabinose
C
+Lev
+Xyl
+Pec
+Cel
CK (LB)

Figure S2

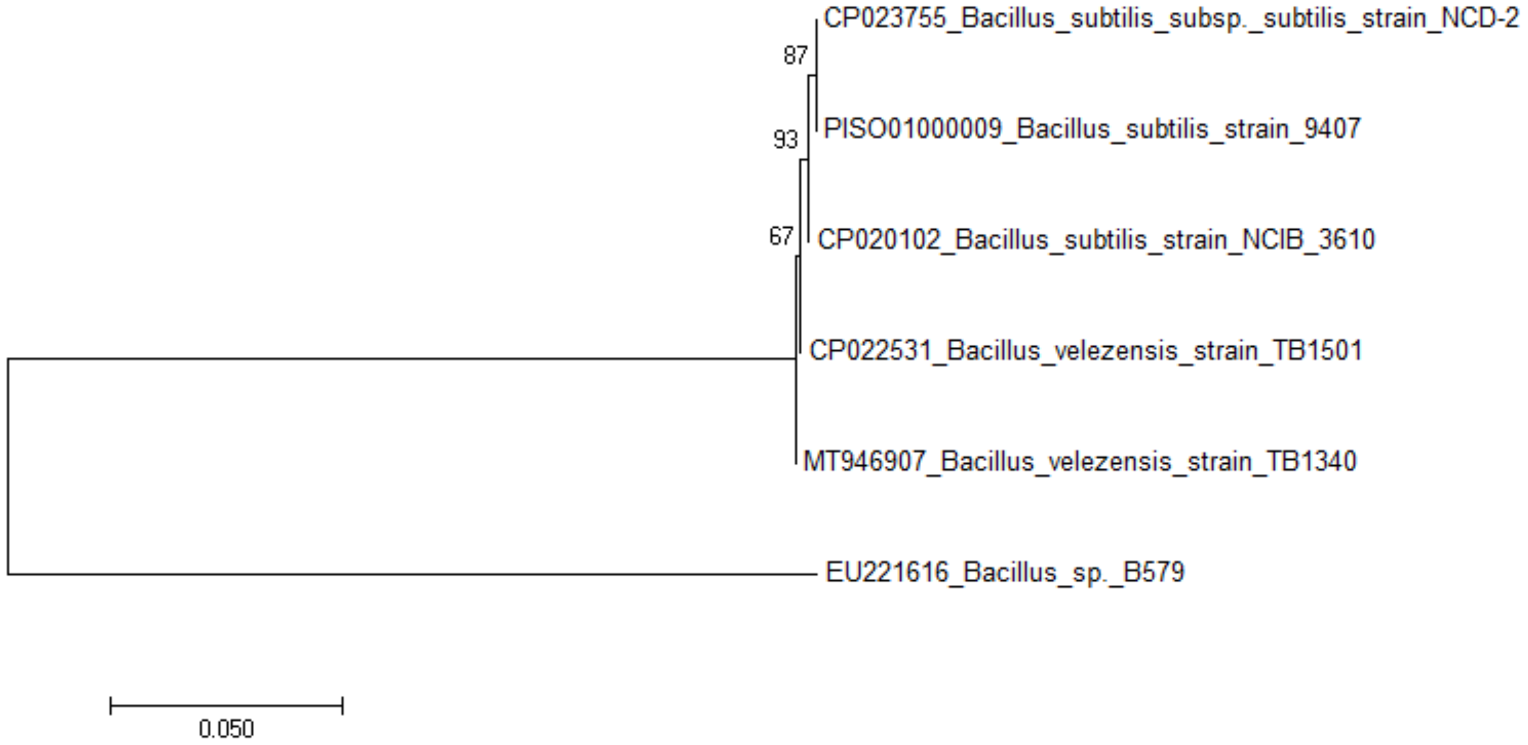
CP023755_Bacillus_subtilis_subsp._subtilis_strain_NCD-2
87
PISO01000009_Bacillus_subtilis_strain_9407
93
CP020102_Bacillus_subtilis_strain_NCIB_3610
67
CP022531_Bacillus_velezensis_strain_TB1501
MT946907_Bacillus_velezensis_strain_TB1340
EU221616_Bacillus_sp._B579
0.050

Figure S3

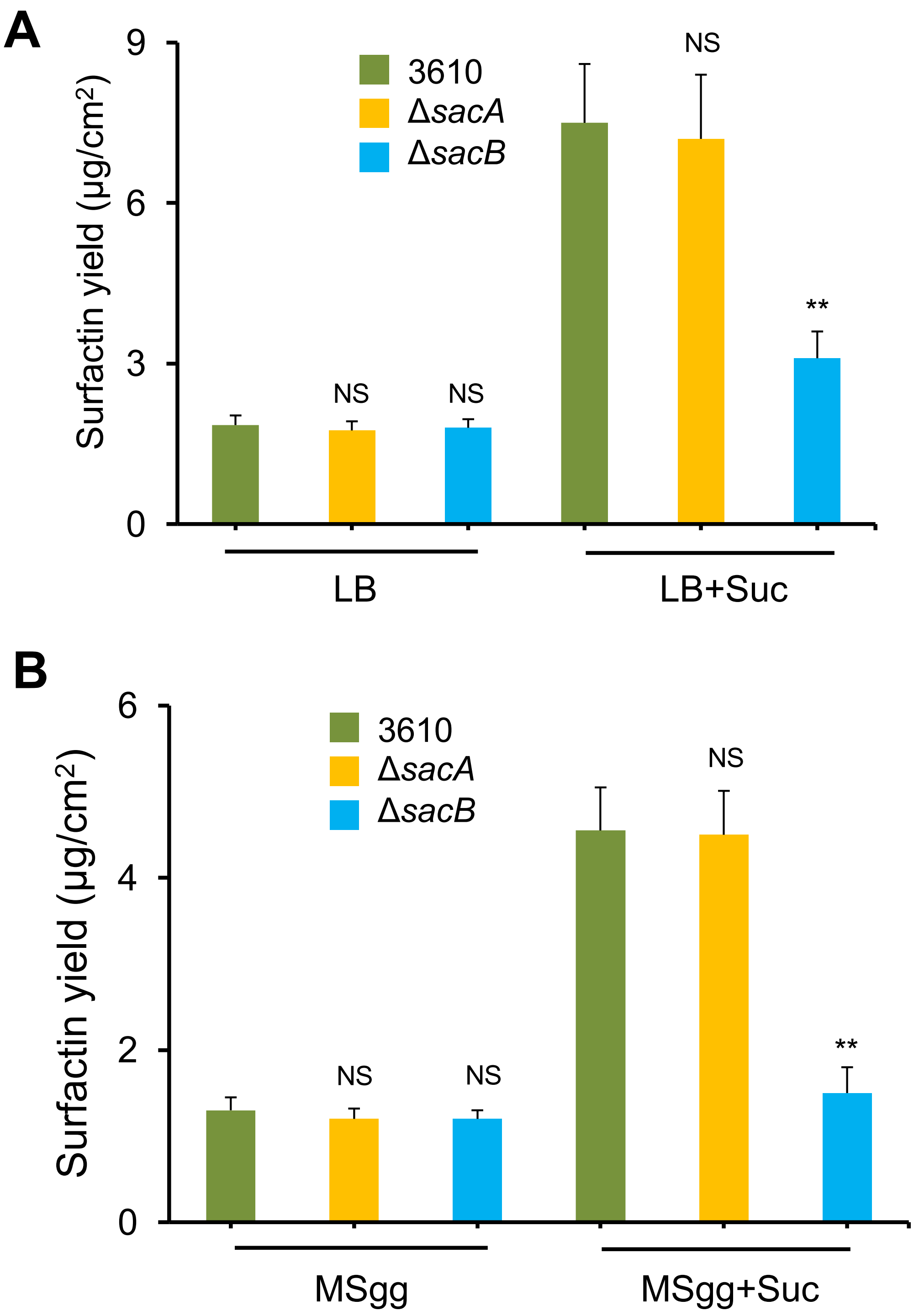
A
Surfactin yield (μg/cm2)
9
6
3
0
3610
ΔsacA
ΔsacB
NS
NS
NS
**
LB
LB+Suc
B
Surfactin yield (μg/cm2)
6
4
2
0
3610
ΔsacA
ΔsacB
NS
NS
NS
**
MSgg
MSgg+Suc

Figure S4

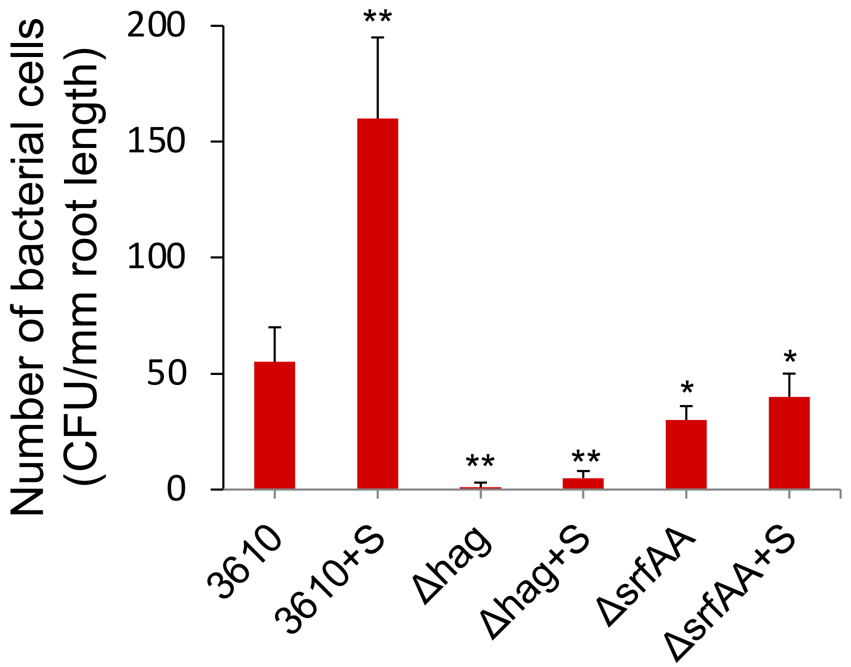

Number of bacterial cells
(CFU/mm root length)
200
150
100
50
0
**
**
**
*
*
3610
3610+S
Δhag
Δhag+S
ΔsrfAA
ΔsrfAA+S

Figure S5

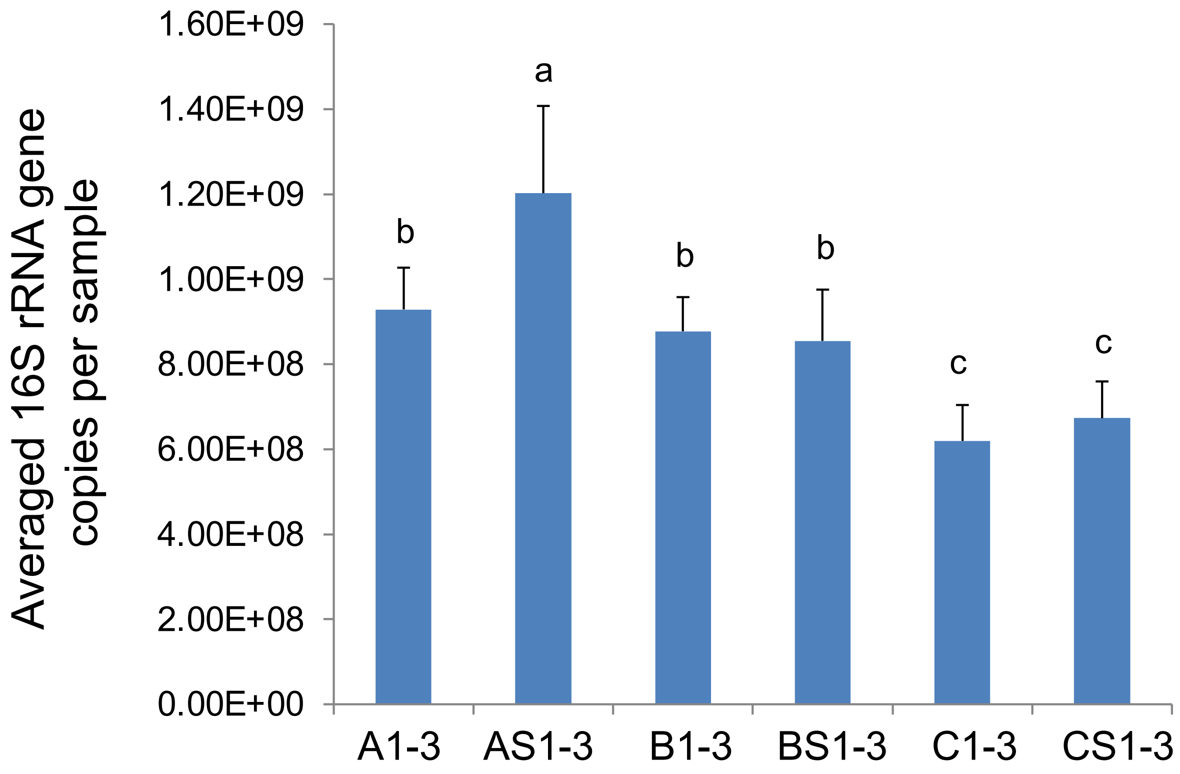
Averaged 16S rRNA gene copies per sample
1.60E+09
1.40E+09
1.20E+09
1.00E+09
8.00E+08
6.00E+08
4.00E+08
2.00E+08
0.00E+00
a
b
b
b
c
c
A1-3
AS1-3
B1-3
BS1-3
C1-3
CS1-3

Figure S6

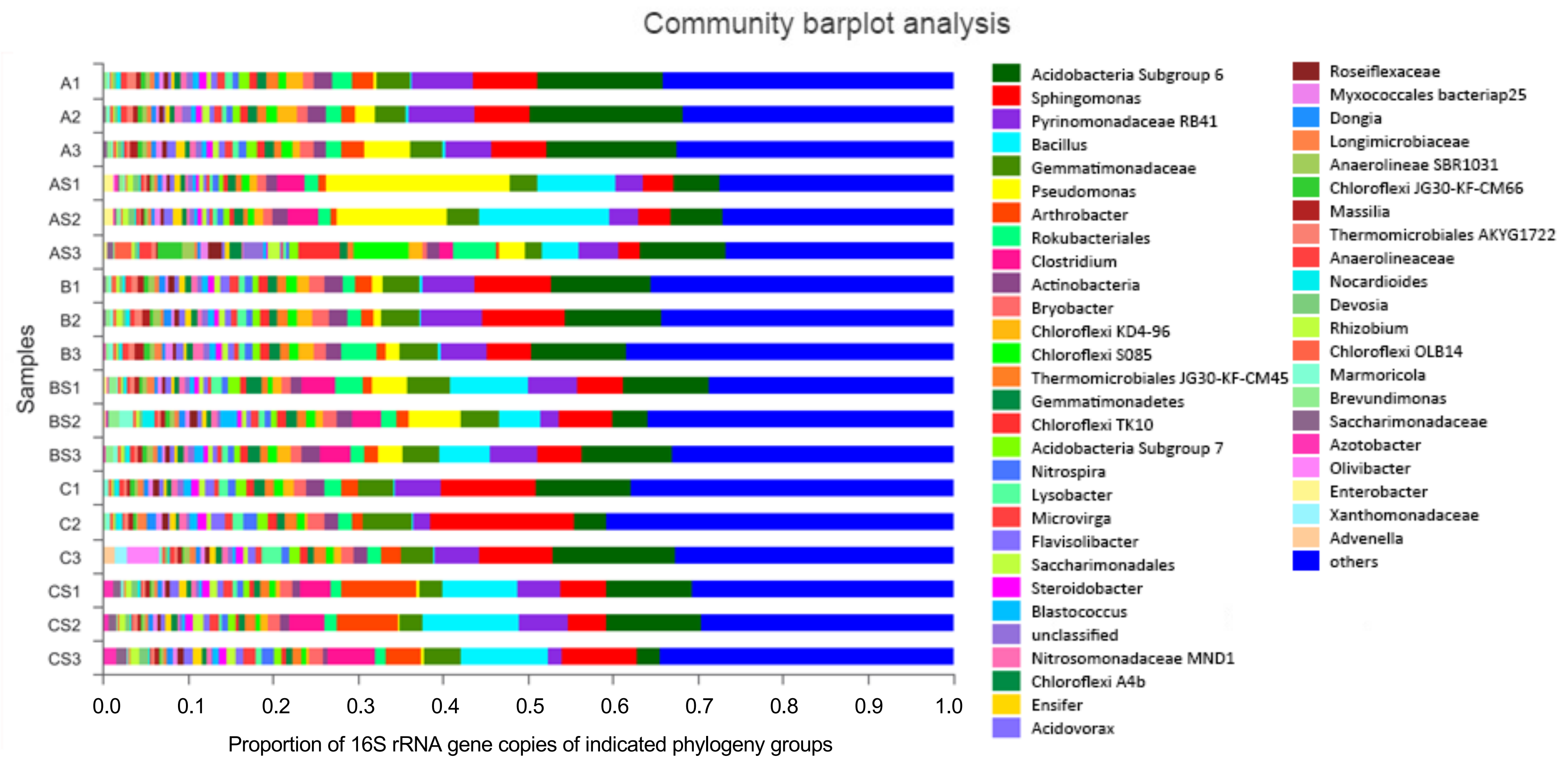
Community barplot analysis
Samples
A1
A2
A3
AS1
AS2
AS3
B1
B2
B3
BS1
BS2
BS3
C1
C2
C3
CS1
CS2
CS3
0.0
0.1
0.2
0.3
0.4
0.5
0.6
0.7
0.8
0.9
1.0
Proportion of 16S rRNA gene copies of indicated phylogeny groups
Acidobacteria Subgroup 6
Sphingomonas
Pyrinomonadaceae RB41
Bacillus
Gemmatimonadaceae
Pseudomonas
Arthrobacter
Rokubacteriales
Clostridium
Actinobacteria
Bryobacter
Chloroflexi KD4-96
Chloroflexi S085
Thermomicrobiales JG30-KF-CM45
Gemmatimonadetes
Chloroflexi TK10
Acidobacteria Subgroup 7
Nitrospira
Lysobacter
Microvirga
Flavisolibacter
Saccharimonadales
Steroidobacter
Blastococcus
unclassified
Nitrosomonadaceae MND1
Chloroflexi A4b
Ensifer
Acidovorax
Roseiflexaceae
Myxococcales bacteriap25
Dongia
Longimicrobiaceae
Anaerolineae SBR1031
Chloroflexi JG30-KF-CM66
Massilia
Thermomicrobiales AKYG1722
Anaerolineaceae
Nocardioides
Devosia
Rhizobium
Chloroflexi OLB14
Marmoricola
Brevundimonas
Saccharimonadaceae
Azotobacter
Olivibacter
Enterobacter
Xanthomonadaceae
Advenella
others

Figure S7

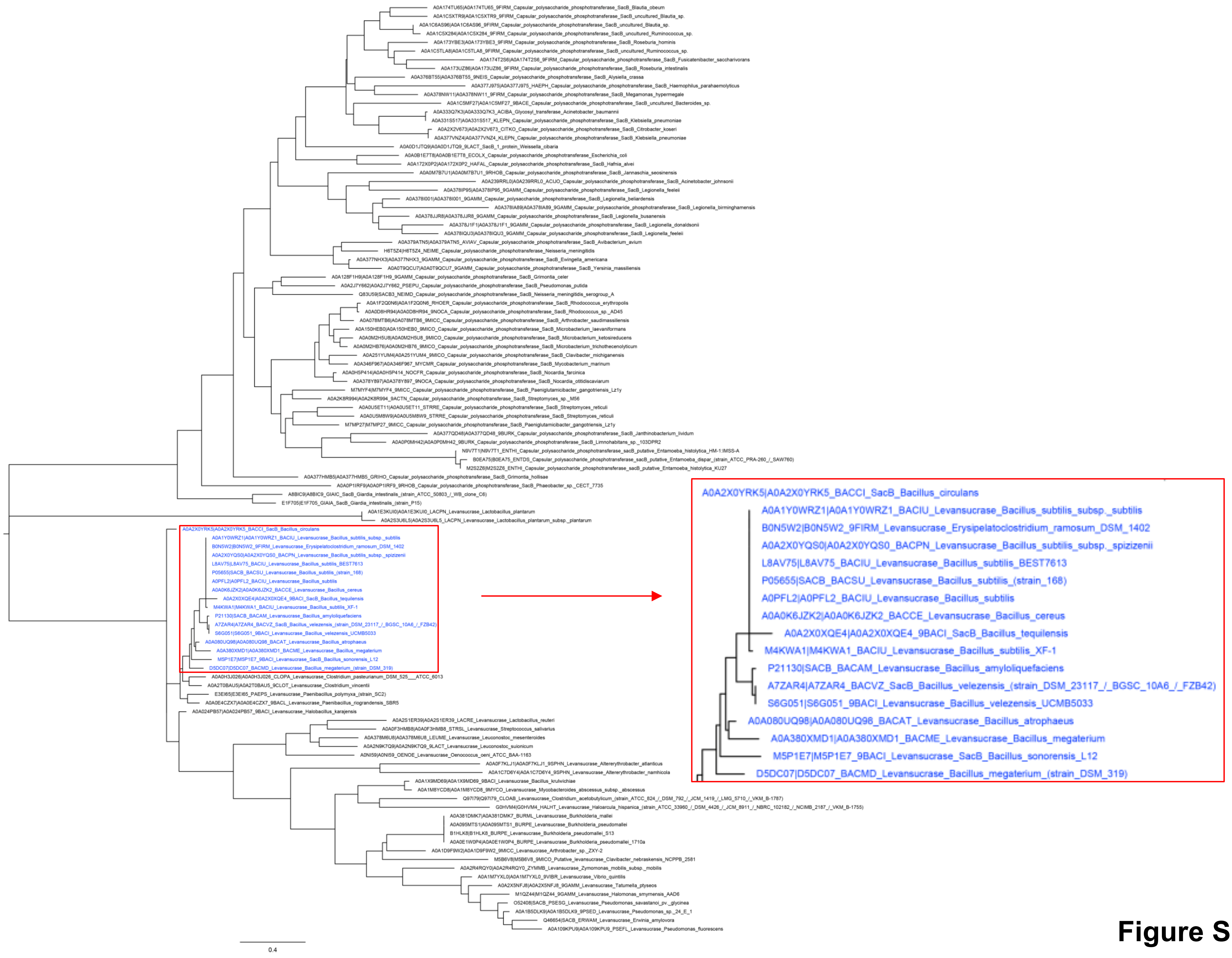

Figure S8

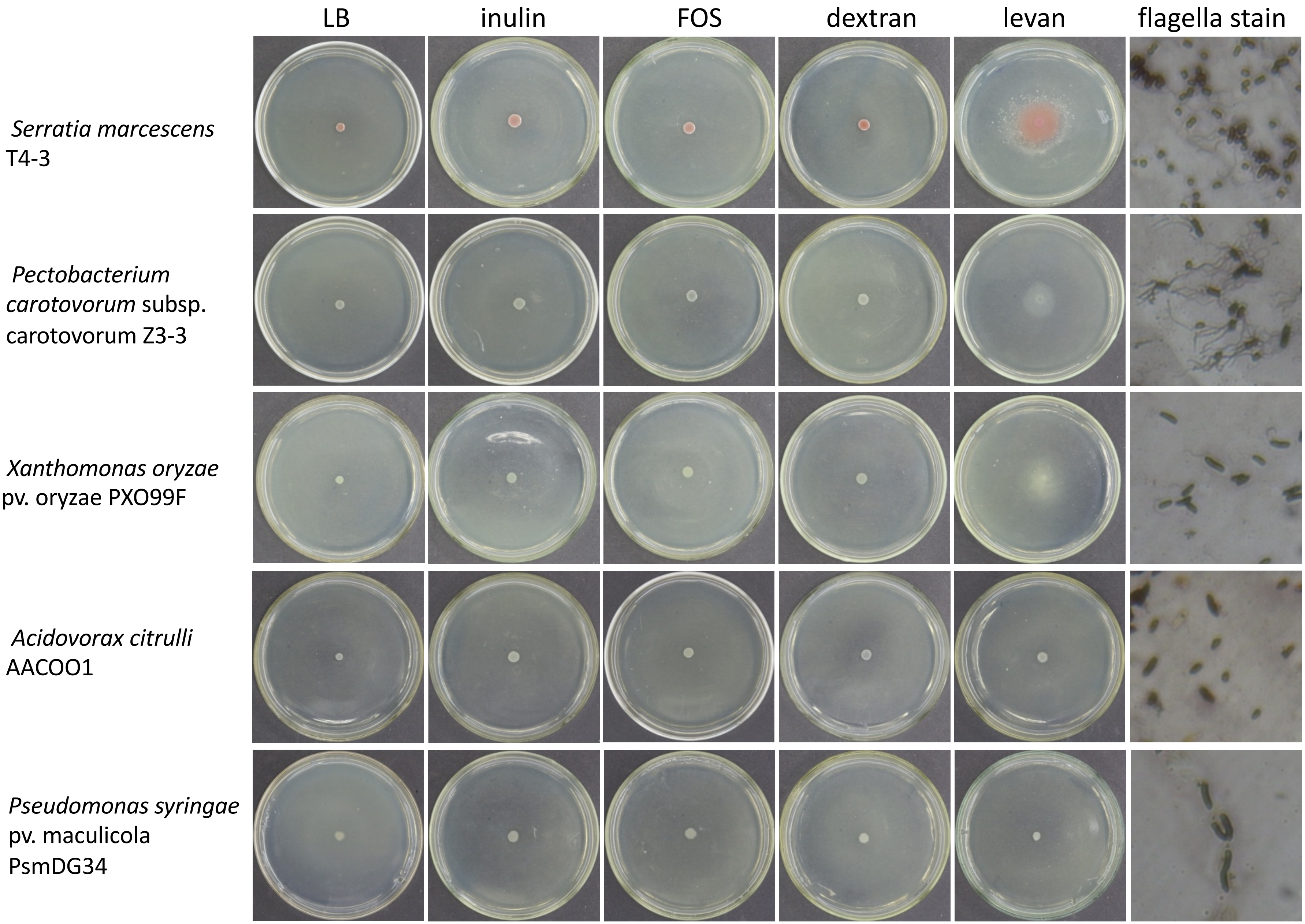
LB
inulin
FOS
dextran
levan
flagella stain
Serratia marcescens T4-3
Pectobacterium carotovorum subsp. carotovorum Z3-3
Xanthomonas oryzae pv. oryzae PXO99F
Acidovorax citrulli AACOO1
Pseudomonas syringae pv. maculicola PsmDG34

Figure S9

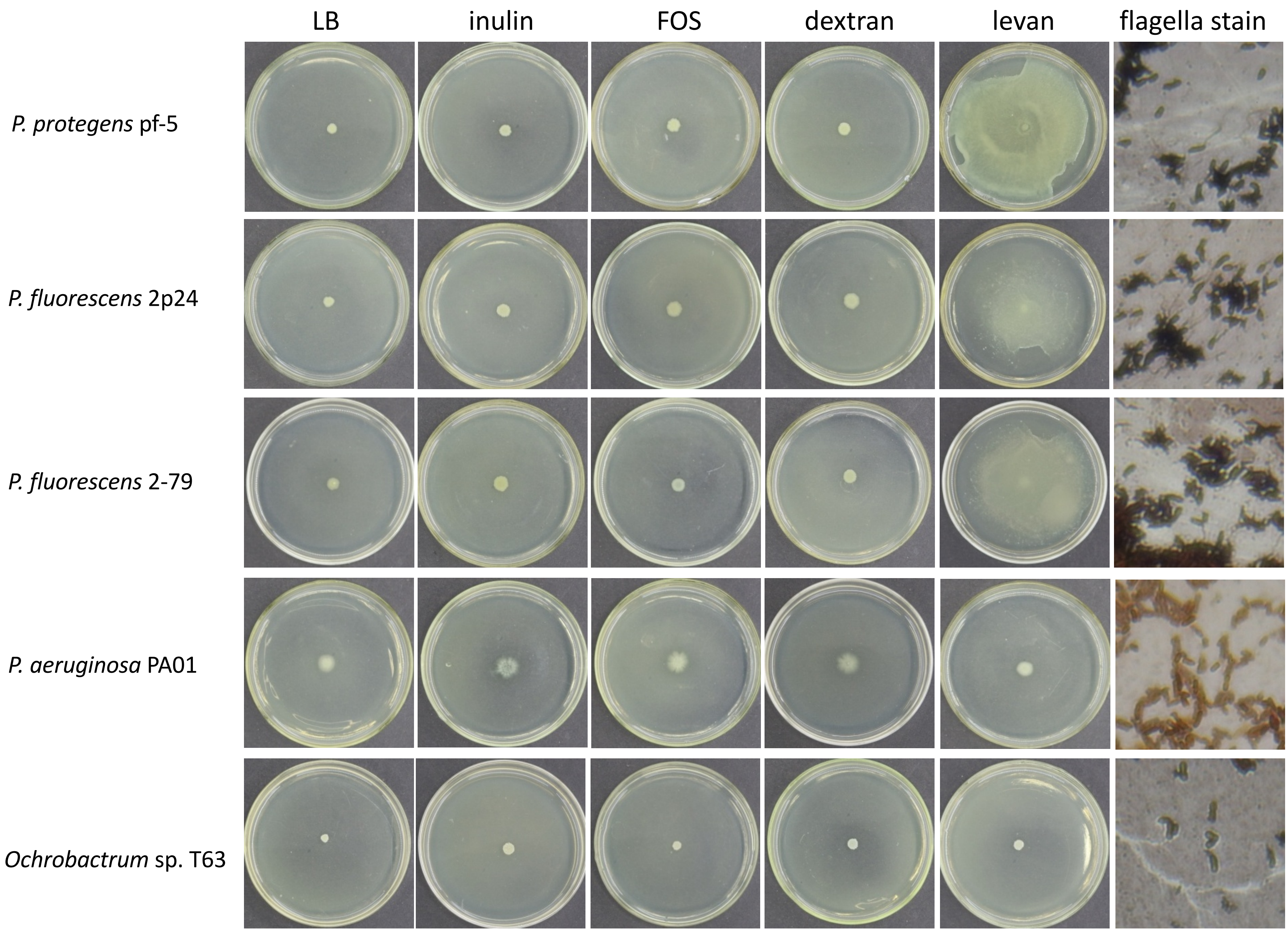
LB
inulin
FOS
dextran
levan
flagella stain
P. protegens pf-5
P. fluorescens 2p24
P. fluorescens 2-79
P. aeruginosa PA01
Ochrobactrum sp. T63

Figure S9
